# Polarization insensitive dual band metamaterial with absorptance for 5G sub-6 GHz applications

**DOI:** 10.1038/s41598-022-12106-7

**Published:** 2022-05-19

**Authors:** Md. Mhedi Hasan, Mohammad Tariqul Islam, M. Salaheldeen M., Sami H. A. Almalki, Abdullah G. Alharbi, Haitham Alsaif, Md. Shabiul Islam, Md. Samsuzzaman

**Affiliations:** 1 Department of Electrical, Electronic and Systems Engineering, Faculty of Engineering and Built Environment, Universiti Kebangsaan Malaysia, 43600 Bangi, Malaysia; 2 Department of Electrical Engineering, Faculty of Energy Engineering, Aswan University, Aswan, 81528 Egypt; 3 Department of Electrical Engineering, College of Engineering, Taif University, P.O. Box 11099, Taif, 21944 Saudi Arabia; 4 Department of Electrical Engineering, Faculty of Engineering, Jouf University, Sakaka, 42421 Saudi Arabia; 5 Electrical Engineering Department, College of Engineering, University of Ha’il, Ha’il, 81481 Saudi Arabia; 6 Faculty of Engineering, Multimedia University (MMU), 63100 Cyberjaya, Selangor Malaysia; 7 Department of Computer and Communication Engineering, Faculty of Computer Science and Engineering, Patuakhali Science and Technology University, Dumki Upazila, Bangladesh

**Keywords:** Metamaterials, Electrical and electronic engineering

## Abstract

A couple ring enclosed circular geometric resonator (CRECGR) based dual-band polarization insensitive metamaterial (MM) with high effective medium ratio (EMR), and excellent absorptance is proposed in this study, which can be utilized as a sensor and absorber in the 5G sub-6 GHz frequency range. A circular geometry-based unique patch has been introduced in the proposed unit cell to achieve high polarization insensitive properties with excellent absorption for the 5G sub-6 GHz spectrum. The distinctive feature of this proposed CRECGR unit cell is its simple and unique structure with a high EMR of 11.13, polarization insensitive up to 180°, and epsilon negative (ENG) properties, including a negative refractive index and near-zero permeability for 5G sub-6 GHz applications. Furthermore, this designed unit cell yields excellent absorption properties with high quality factor. The designed MM unit cell is fabricated on low loss Rogers RT5880 printed media with an electrical dimension of 0.089λ × 0.089λ × 0.017λ. The performance of the designed CRECGR metamaterial is determined using Computer Simulation Technology (CST), Advanced Design Software (ADS), and measurements. The CRECGR unit cell offers dual resonances at 3.37 GHz and 5.8 GHz, covering the 5G sub-6 GHz band with ENG, near-zero permeability and negative index. The polarization insensitive properties of the unit cell were also investigated for maximum angle of incidence, which confirmed the identical response. The simulated outcome is verified by experiment with excellent accordance. Moreover, the unit cell performance with a complete backplane is explored, noting a maximum absorption of 99.9% for all normal and oblique incidence waves, suitable for sensing and antenna systems. In addition, the suggested unit cell sensing performance is evaluated using the permittivity-based sensing model. The proposed MM outperforms recent related studies in terms of polarization insensitivity up to 180°, high insensitive absorptivity, high EMR, and sensing applications. These features prove that the proposed CRECGR metamaterial is perfect for 5G Applications.

## Introduction

Metamaterials are artificially engineered media that can generate macroscopic properties when they interact with waves, gained from geometric structure, not a composition. Researchers from all around the world have focused their efforts on metamaterials, which exhibit unique properties like artificial plasmas, negative permeability, and negative refractive index, for use in a variety of future wireless communication applications. These applications encompass antenna performance enhancement^1–5^, specific absorption rate (SAR) reduction^6^, absorber^7,8^, sensing^9,10^, energy harvesting^11,12^, lensing^13^, future communications, remote aircraft and solar energy management^14–17^, and so on. Double negative metamaterials (DNG) and single negative metamaterials (SNG) are two types of metamaterials that are frequently designed using a split-ring resonator (SRR)^18,19^. Moreover, the SNG metamaterials can be classified as epsilon negative (ENG) (permittivity negative) or mu negative (MNG) (permeability negative). The resonance frequency in the microwave or millimeter wave bands is governed by the geometrical structure of the SRR. In the 5G spectrum, the proposed metamaterial could be an attractive prospect for 5G wireless communications applications. The 5G frequency spectrum is split into two parts: sub-6 GHz, which is utilized in both 4G and 5G communication, and millimeter wave, which is exclusively used in 5G systems^20^. Due to the rapid advancement of technology in the future wireless communication system, scientists and engineers have focused their attention on these frequency bands. Thus, the focus of this study is on developing a compact metamaterial structure with excellent absorptance properties for sub-6 GHz 5G applications.

A hexagonal-shaped SRR based MM is proposed in^21^ for dual-band microwave applications with DNG and absorptance properties. However, this unit cell exhibits low EMR value and poor absorption at a higher resonance. Moreover, the multiband MM unit cell has been presented in^22,23^ for microwave applications with a higher covering band but have low EMR values. In 2021, a mender line based ENG MM unit cell is proposed for C band application with an EMR of 10.60 but have only one resonance^9^. A complementary SRR-based single band MM unit cell with a low EMR value and petite size has been reported in another work^24^. A concentric crossed-line based ENG MM structure is highlighted in^25^ for multiband microwave applications. In this vein, researchers investigated a left-handed sandwiched MM with customizable characteristics for microwave applications^26^. The article^27^ describes an asymmetric SNG metamaterial structure with sensing applications in the microwave spectrum and offers more than 90% absorption with good selectivity. However, owing to the polarization effect of the asymmetric structure, it cannot be used for oblique incident waves. A bilaterally coupled epsilon negative dual-band metamaterial with near-zero permeability and refractive index has been reported in^28^ for X and C band communication systems. The resonance frequency is tuned in this article using bilateral connected metal width. The authors presented a single negative MM unit cell for microwave sensing applications in^29^, which is based on a modified SRR, operating at a frequency of 140 GHz. The authors of^30^ introduce an inductively controlled metamaterial for multiband antenna gain improvement applications. Additionally, when using a copper backplane, this MM demonstrates absorption and selectivity but have poor absorption at 8.48 GHz frequency. A multiband ENG metamaterial with a near-zero index and high value of EMR is designed and investigated in^31^ for antenna gain improvement. The absorptance properties, as well as unit cell polarization parameters, are not shown in this research. A polarization independent dual-band metamaterial has been devised for wireless communication using a Rogers RT6002 dielectric substrate^32^. This MM structure has a low EMR and simple construction while also considering a maximum polarizing angle of incidence of 45°. The study in^33^ presented a polarization-sensitive dual-band perfect metamaterial absorber for Ku-band applications with maximum absorption of 99.87%. In^8,34,35^, a variety of absorbers are developed and explored that employ metamaterial structures for microwave and millimeter wave absorption. Among these, the authors of^34^ presented a perfect absorber based on MM with a 90% absorption bandwidth for use in the k band. Further, this article addresses the sensing capability of the suggested MM absorber without experimental confirmation. On the other hand, a co-polarized perfect metamaterial for Wi-Fi applications is devised in^35^, focusing on metamaterial equivalent circuit analysis for desired frequency applications. This paper demonstrated maximum 99% absorption with high EMR value. The author of^8^ designed a crescent shape wide-angle MM absorber for Wi-Fi networks with a maximum absorption of 99%, where the absorption varies 99% to 93% depending on the angle of incident. A fractal wideband absorber based on an E-shaped split ring resonator for a 5G millimeter wave communication application with a very large unit cell structure is reported in^36^. Although it covers a broad frequency range in the millimeter wave spectrum, it has an absorptivity of around 80% when waves are incident at a normal angle. Using the 3D printing approach, the researchers in^37^ developed a polarization independent water metamaterial with absorption of over 90% for ultra-broadband applications.

Although there have been a few publications proving the compact metamaterial structure with good EMR^7–9,21–25,27,29,31,32,34,36^, no articles for a 5G sub-6 GHz spectrum operating high polarization insensitive metamaterial resonator with a small size, high EMR and perfect absorber qualities have been reported. In this study, a novel high polarization insensitive metamaterial with excellent absorption and sensing qualities has been introduced for 5G sub-6 GHz communication systems. The resonator of the designed CRECGR metamaterial encloses a circular geometric pattern within three square split coupled rings covering the 5G sub-6 GHz frequency spectrum. The unique feature of this metamaterial is its simple and novel architecture, which has a high EMR of 11.13 and is polarization insensitive up to 180° incident angle. Moreover, it extends outstanding absorption properties of 99.9% for both normal and oblique incidence angles as well as a higher quality factor (Q-factor). The absorption in TE and TM mode is identical and insensitive up to 90° oblique incidence angles. Additionally, it possesses ENG features with near-zero permeability and a negative refractive index. The equivalent circuit of the suggested unit cell is developed in ADS, and the performance is verified against CST simulation findings. The proposed prototype is fabricated on a widely used and low loss Rogers RT5880 printed substrate, which possesses excellent electrical properties and chemical resistance. The experimental and numerical findings confirm that the developed prototype is suitable for 5G sub-6 GHz applications. In addition, the developed unit cell performance has been compared with existing relevant published prototypes and found it excellent in terms of design structure, broad polarization insensitivity, high insensitive absorptance and sensing applications. The most important goal of this research is to design a novel metamaterial in the desired 5G frequency range with high polarization insensitivity and perfect absorptance properties, as well as a miniaturized structure with a high EMR for sensing and antenna system applications. Following the introduction, the second section addressed the unit cell's design procedure and simulation technique. The third section illustrates the effective parameters extraction approach and the detailed performance analysis of the unit cell and array structure presented in section four. Section five discusses a succinct investigation of the surface current density, as well as the electric and magnetic field patterns, while section six and seven goes into the equivalent circuit (EC) model and experimental investigation of the resonator. The eighth and ninth sections examined the suggested MM's performance as an outstanding absorber and provided experimental confirmation. Section ten and eleven contains MM sensing applications and a performance comparison table with related current works, as well as a summary of the study in section twelve.

## Unit cell geometry design and simulation procedure

**Figure 1:**
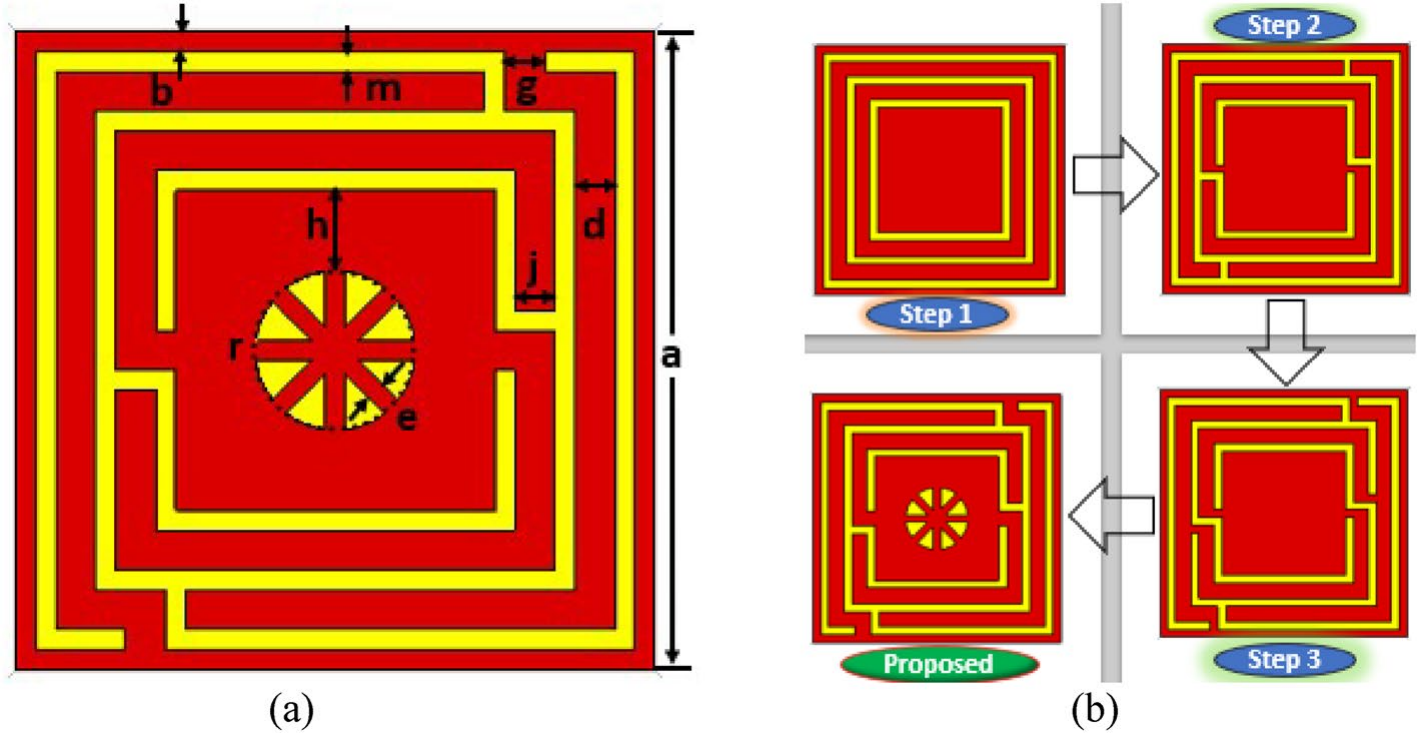
(**a**) The detailed unit cell patch structure with dimension. (**b**) Design evaluation of the proposed MM structure (CST STUDIO SUITE 2019, https://www.3ds.com/products-services/simulia/products/cst-studio-suite)^39^.

**Figure 2:**
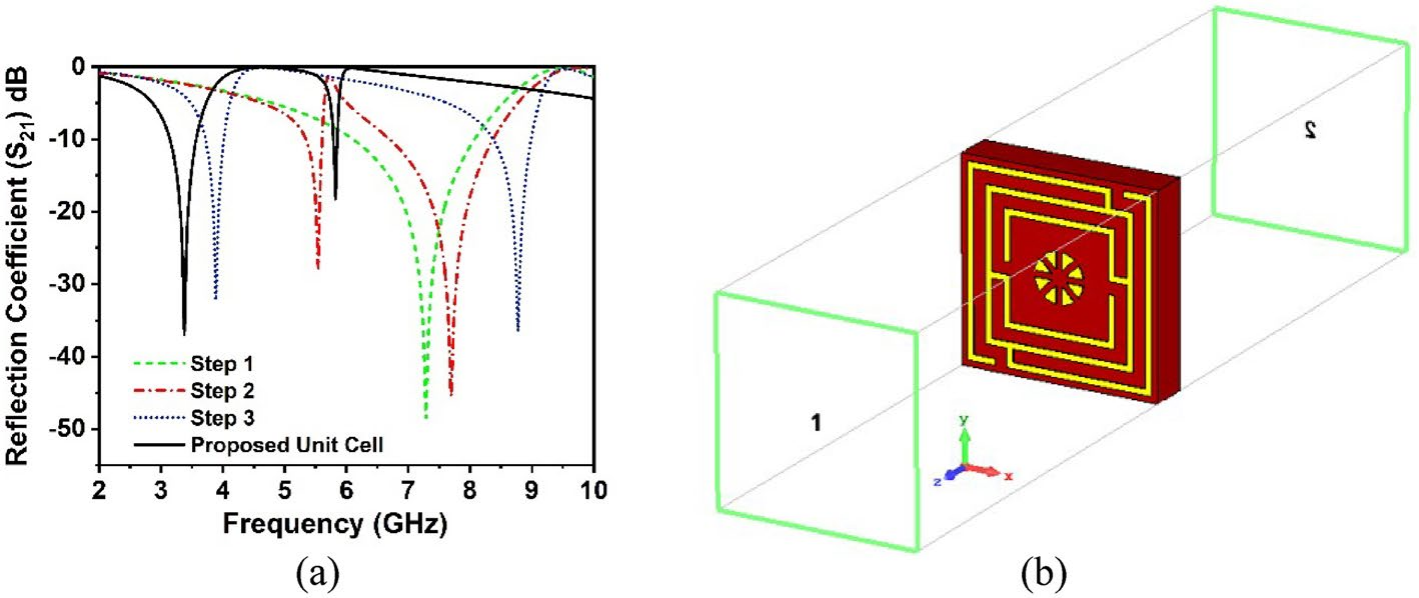
(**a**) Reflection coefficient spectra for different design phases. (**b**) CST Simulation arrangement (CST STUDIO SUITE 2019, https://www.3ds.com/products-services/simulia/products/cst-studio-suite)^39^.

The proposed unit cell geometry encloses a center circular geometric pattern within the square split coupled ring resonator, which is an improved version of the traditional split ring resonator (SSR). The unit cell size is quite small in relation to the wavelength of the resonance frequency, which fulfill the metamaterial’s sub-wavelength standards^38^. The proposed metamaterial is distinguished by its compact size, high EMR, polarization insensitive, absorptance properties, and potential to substantially alter the resonance frequency within the sub-6 GHz frequency spectrum for 5G applications. The proposed MM structure, as well as the dimensions of the different splits and segments, are illustrated in Fig. 1a. The suggested unit cell is realized on Rogers RT5880 dielectric printed media with an electrical dimension of 0.089λ × 0.089λ × 0.017λ at the 3.37 GHz resonance frequency where the dissipation factor and dielectric constant are 0.0009 and 2.2, respectively. Rogers RT 5880 is a well-known and reliable material consisting of lightweight PTFE with random microfiber glass reinforcing. It exhibits homogeneous electrical properties across a large frequency range with very low moisture absorption and low outgassing, making it well suited for use in high-humidity conditions. Moreover, it has the lowest dielectric loss, the highest chemical resistance, and the lowest electrical loss, making it suitable for 5G and beyond high frequency or broadband applications. In addition, it can be easily cut, sheared, and machined into shape, which makes it a convenient material for manufacture. As this proposed design demands excellent electrical properties and advanced performance at the 5G sub-6 GHz operating frequency, Rogers RT5880 is chosen, which supports great performance in the 5G sub-6 GHz frequency range. The split-ring resonator (SSR) with a 0.035 mm copper thickness is used to fix the resonant frequency. The split gaps, connecting strips, ring position and the center circular geometric pattern are determined by the trial-and-error process to obtain the desired frequency band in the 5G spectrum. The optimal resonator is composed of three-square rings and one circular geometric design in which the width of the splits and connecting metals modulate the resonances. Moreover, the central circular pattern significantly switches the operating frequencies of the resonator. The unit cell design evaluation is presented in Fig. 1b and the various optimized dimensions of the proposed MM structure are illustrated in Table 1. In design step 1, the three-square rings are spaced similarly and have the same metal width on one side of the substrate, while the other side is copper-free, as indicated in Fig. 1b. These rings interact as an inductor that exhibits a single resonance at the frequency of 7.3 GHz, as shown in Fig. 2a. In the following design, the first ring is connected to the second ring using a metal strip, and the second is similarly connected to the third square ring by introducing two splits in the third ring. Incorporating the splits and connecting metal offers two resonances at 5.6 GHz and 7.6 GHz with the previous resonance shifting, as illustrated in Fig. 2a. The resonances and shifting characteristics are caused by the capacitive and inductive effects, as well as mutual coupling effects between the rings. The splits in the first and second square rings are appended in an optimal fashion to achieve the desired frequency bands of 3.8 GHz and 8.7 GHz, respectively, as indicated in the third design stage. In this step, the first resonance frequency is significantly reduced to a lower value, while the second resonance is moved to a higher value. Finally, a circular ring with an optimal geometric layout is proposed to cover the desired band of 3.37 GHz and 5.8 GHz for 5G sub-6 GHz applications, as depicted in Fig. 2a. The designed circular geometrical pattern demonstrates the effect of shifting both resonances, particularly the second resonance, which is considerably suppressed.

**Table 1:** Parametric values of the proposed MM structure.

Parameter	Size (mm)	Parameter	Size (mm)	Parameter	Size (mm)
*a*	8	*g*	0.5	*h*	1
*b, e, m*	0.25	*d, j*	0.5	*r*	3.14

**Figure 3:**
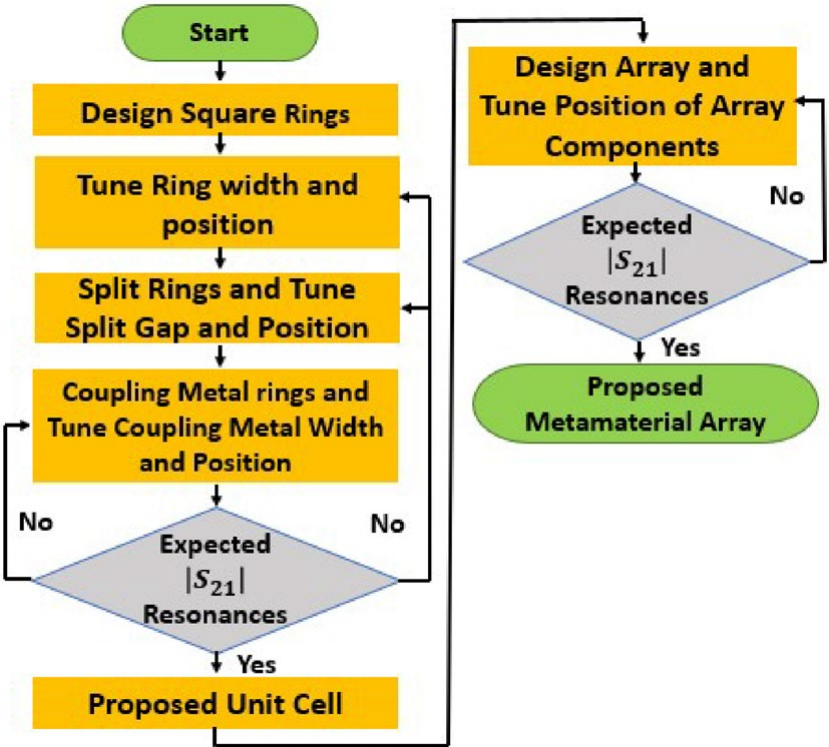
The flowchart for designing and optimizing the suggested MM structure.

The numerical simulator CST microwave studio suite, 2019, with a frequency-domain solver, has been used to conduct the proposed MM unit cell and array simulation. The CST simulation arrangement of the proposed MM structure is illustrated in Fig. 2b, where transmitting and receiving ports are aligned in the Z-axis and excite the designed MM structure to extract the characteristics that often do not exist in natural materials. In order to meet the boundary requirements, electric and magnetic fields are utilized in the X- and Y-axes. The perfect electric conductor (PEC) condition has two distinct behaviors: first, it acts perpendicular to the electric field with unit cell bottom and topsides, and second, it behaves perpendicular to the magnetic field with unit cell front and rear sides. The open add distance of the boundary conditions is determined using the formula, ${2D}^{2}/\lambda$, where D is the unit cell diameter $\lambda$ is the wavelength in free space, which assists in analyzing the electric field in terms of near field zone^21^. The simulation frequency is adjusted to between 2 and 10 GHz, and a tetrahedral mesh is used to simulate unit cells and arrays. Figure 3 illustrates the comprehensive design approach and optimization for the suggested MM geometry. The flowchart summarizes the principal design parameters and their contribution to the properties of MM S_21_.

## Metamaterial effective parameters extraction technique

The effective media characteristics of the proposed MM unit cell and array can be retrieved from the S-parameters using the CST post-processing approach, including the permittivity, permeability, refractive index, and effective impedance. The CST simulator extracted the effective parameters of the unit cell and array structure from the scattering parameters utilizing a well-established robust retrieval technique. The refractive index and impedance can be approximated using the following transmission and reflection coefficient equations, which are explained by the robust retrieval approach^40^.

1 $${\text{Reflection}}\,{\text{coefficient}},\quad {\text{S}}_{11} = \frac{{R_{01} \left( {1 - e^{{i2nk_{0} d}} } \right)}}{{1 - R^{2}_{01} e^{{i2nk_{0} d}} }}$$

2 $${\text{Transmission}}\,{\text{coefficient}},\quad {\text{S}}_{21} = \frac{{(1 - R^{2}_{01} ) e^{{ink_{0} d}} }}{{1 - R^{2}_{01} e^{{i2nk_{0} d}} }}$$

where $$R_{01} = \frac{z - 1}{{z + 1}}.$$

The impedance and refractive index are calculated by reversing Eqs. (1) and (2):

3 $${\text{Impedance}},\quad z = \pm \sqrt {\frac{{\left( {1 + S_{11} } \right)^{2} - S^{2}_{21} }}{{\left( {1 - S_{11} } \right)^{2} - S^{2}_{21} }}}$$

4 $${\text{and}}\,{\text{the}}\,{\text{refractive}}\,{\text{index}},\quad {\text{n}} = \frac{1}{{K_{0} d}}{ }\left\{ {\left[ {\left[ {\ln \left( {e^{{ink_{0} d}} } \right)} \right]^{{^{\prime\prime}}} + 2m\pi } \right] - i{ }\left[ {\ln \left( {e^{{ink_{0} d}} } \right)} \right]^{^{\prime}} } \right\}$$

Here, the real and imaginary elements of the operator are denoted by (.)' and (.)" correspondingly, while the integer number m correlates to the real refractive index. The permittivity and permeability are calculated using the equations, which are based on the impedance and refractive index.

5 $${\text{Permittivity}},\quad \varepsilon { } = { }n/z$$

6 $${\text{and}}\,{\text{permeability}},\quad \mu = nz$$

The metamaterial structure might be modelled as a medium with an electromagnetic (EM) mesh array that is defined by the plasma frequency.

## Prototype performance

### Unit cell performance analysis

**Figure 4:**
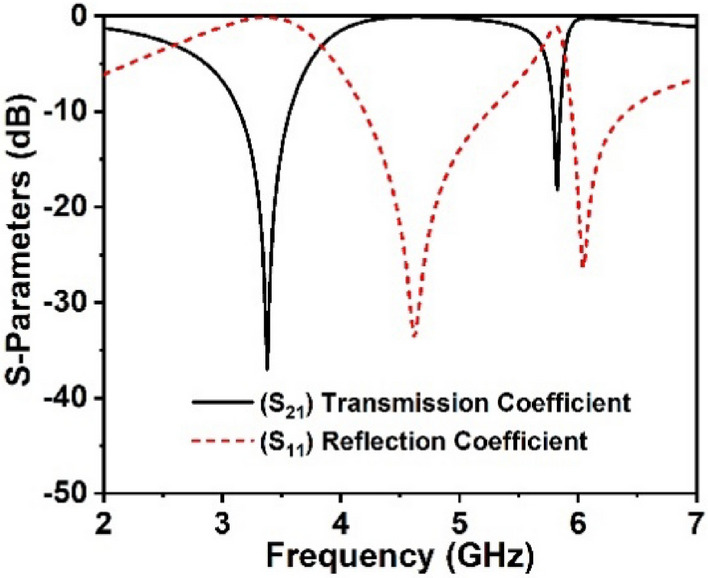
S-parameters of the suggested MM structure.

The performance of the unit cell prototype and array arrangement is investigated employing CST simulator, and the simulation results are confirmed by the experimental results. The transmission and reflection coefficient of the proposed unit cell is presented in Fig. 4, which offers two resonances at 3.37 GHz and 5.8 GHz in the 5G sub-6 GHz frequency band. These resonances possess − 10 dB passband ranges of 3.1–3.6 GHz and 5.76–5.9 GHz, respectively, with magnitudes of − 37 dB and − 18.5 dB. Figure 4 clearly demonstrates that each resonance of S_11_ happens following the resonance of S_21_; hence, the electrical resonances are ensured.

**Figure 5:**
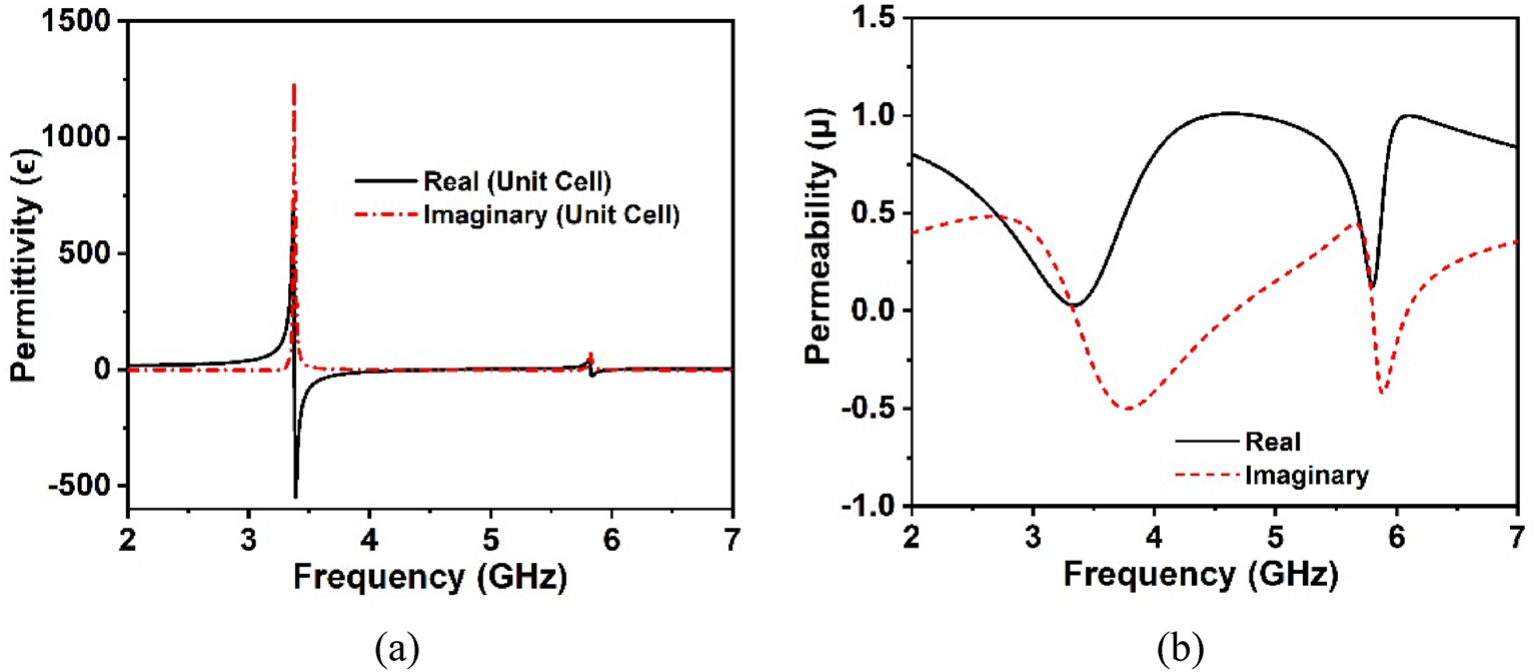
(**a**) Permittivity and (**b**) permeability (CST post processing approach) of the proposed unit cell at operating bands.

**Figure 6:**
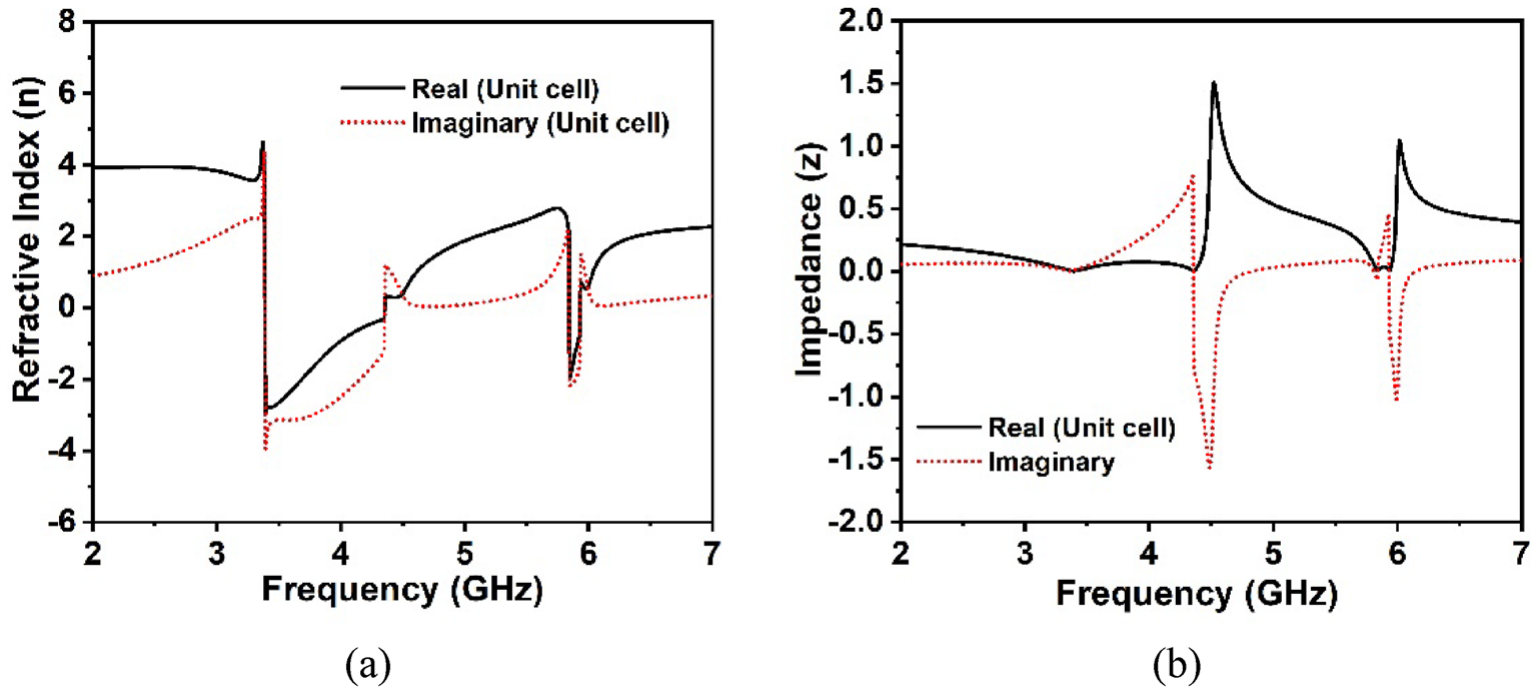
(**a**) Refractive index and (**b**) input impedance (CST post-processing approach) of the proposed unit cell at operating bands.

Figure 5 illustrates the effective relative permittivity and permeability of unit cells retrieved from the CST post-processing module using a robust retrieval approach. It is noticeable that the permittivity values of the unit cell fluctuate between positive and negative values, however, the effective permeability solely displays positive values throughout the desired frequency band, confirming that the suggested unit cell possesses epsilon negative characteristics. Moreover, as seen in Fig. 5a, the highest negative permittivity values arise vicinity of the S_21_ resonance frequencies, whereas the least permeability values occur at the resonance frequencies shown in Fig. 5b. In Fig. 6a, the CST retrieval values for the effective refractive index of the proposed unit cell are demonstrated beside the frequency. It is worth noting that the suggested MM supports the presence of a negative refractive index at 3.37 GHz and 5.8 GHz, sequentially, in the S_21_ spectrum. Furthermore, the real negative peak values of the refractive index exist inside the spectrum of permittivity and permeability. These features of the proposed MM could be applied to antenna intention to enhance bandwidth, gain, and performance^30,41^. Figure 6b depicts the effective impedance values of the proposed unit cell, where the real values are positive, thus the suggested unit cell is a passive medium. Table 2 summarizes the extracted distinctive features of the designed unit cell structure’s effective parameters.

**Table 2:** Characteristics of the suggested MM structure's effective parameters.

Parameters	Frequency range (GHz)	Near-zero/negative region	Bandwidth threshold
*S*_*21*_	3.1–3.6 and 5.76–5.8		$S_{21} < - 10\,{\text{dB}}$
$\varepsilon_{r}$	3.37–4.5 and 5.8–5.95		$\varepsilon_{r} < 0$
$\mu_{r}$	3.37 and 5.8	0.02 and 0.13 (real)	$\mu_{r} < 0$
*n*	3.39 and 5.81	− 2.88 and − 2.06 (real)	$n < - 1.5$

### Array analysis

**Figure 7:**
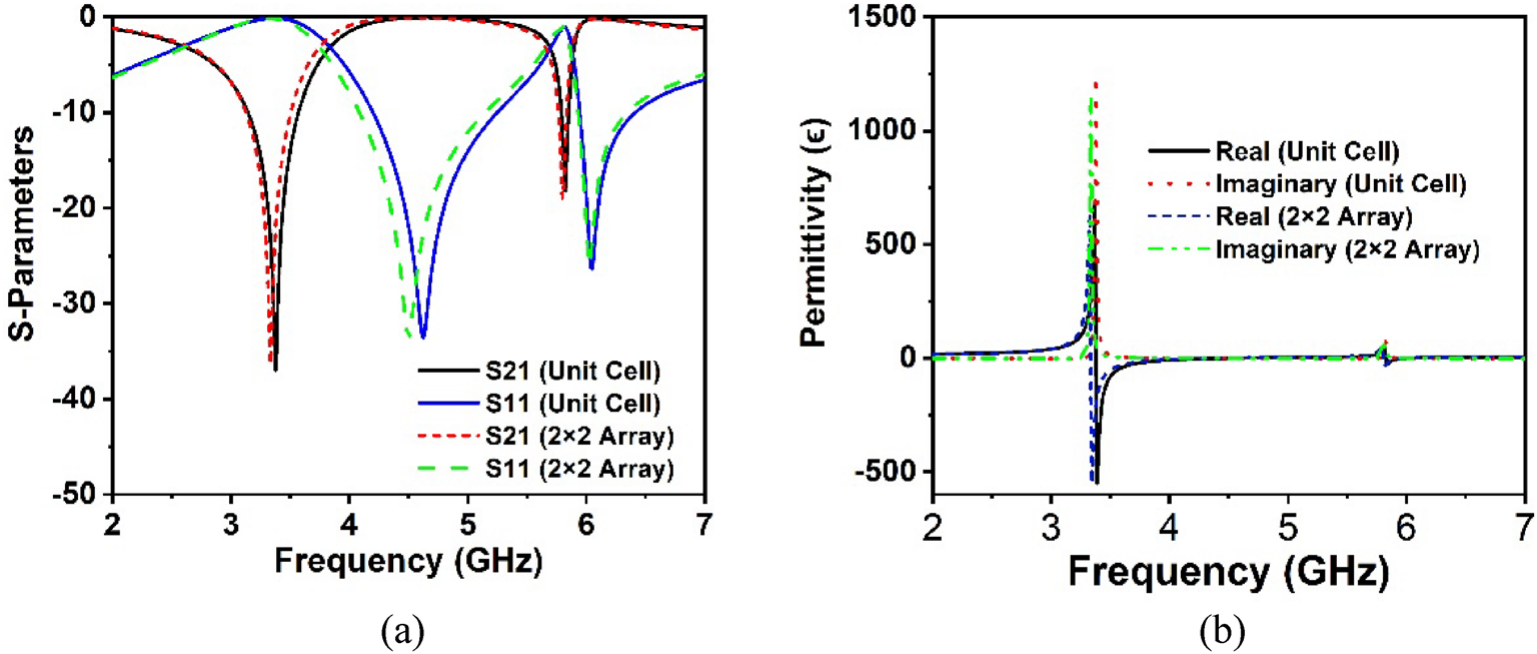
Comparison plots of (**a**) scattering parameters and (**b**) CST extracted permittivity for unit cell and 2 × 2 array.

**Figure 8:**
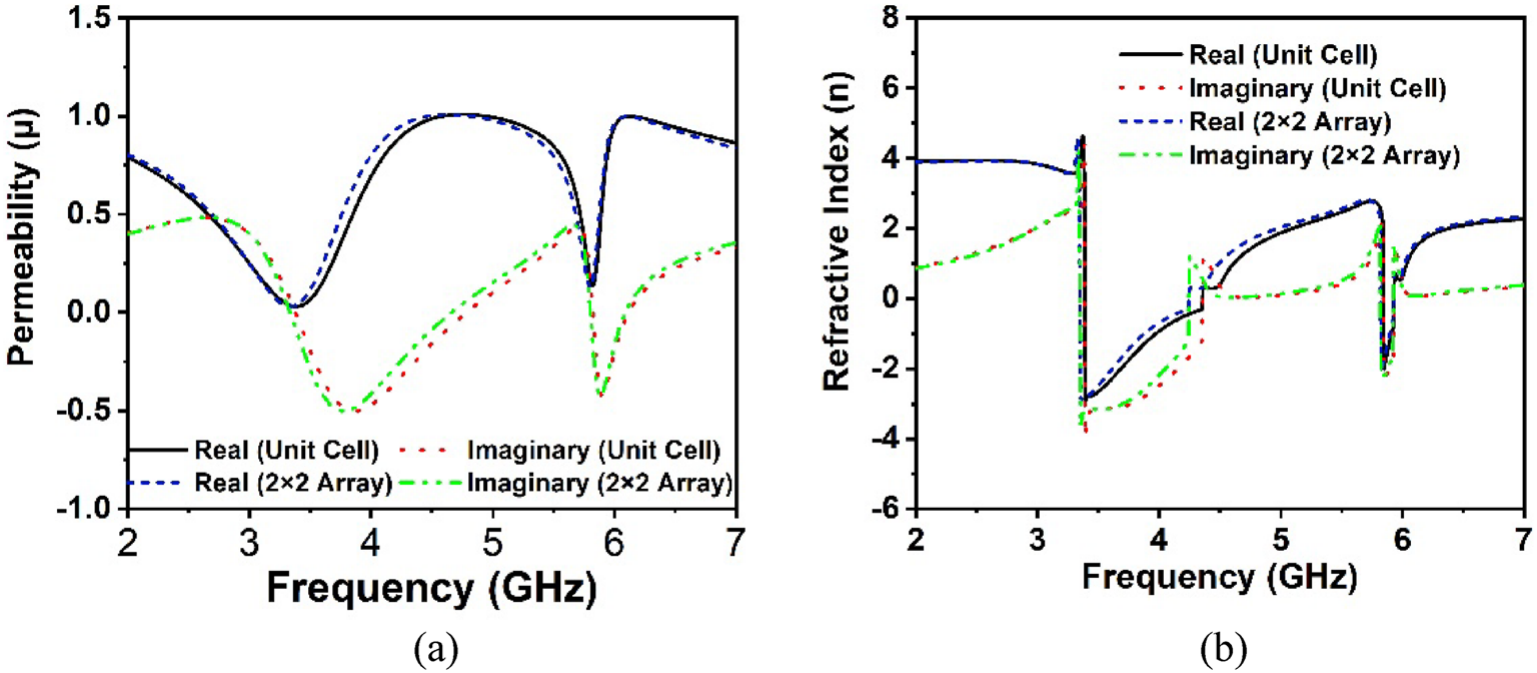
Comparison plots of CST extracted (**a**) permeability and (**b**) refractive index for unit cell and 2 × 2 array.

The performance of the 2 × 2-unit cell array is investigated with a similar simulation setup of the single unit cell, as most applications employ an MM array rather than a unit cell. Figure 7a illustrates the reflection and transmission coefficients of the unit cell array in comparison to the unit cell response. It is important to highlight that the array's S_21_ resonance frequencies are almost identical to unit cell resonances, with the exception that the array's S_11_ lower resonance frequency deviates somewhat from the unit cell response, while the upper resonance remains consistent. As a consequence, the effect of mutual coupling between the array components is negligible in our proposed MM structure. The CST extracted effective parameters of the array including such permittivity, permeability and refractive index, are illustrated in Figs. 7b and 8a,b, respectively, in comparison to the unit cell results. The array findings are amazingly closer to the proposed unit cell, as seen in these Figs. 7b and 8a,b, with just a little difference owing to the negligible mutual coupling effect. In addition, the real permeability show near to zero values at both resonances, which confirms the applications in the antenna design, where it can tailor the antenna radiation pattern to enhance the antenna gain and directivity^41^.

### Polarization insensitivity analysis

**Figure 9:**
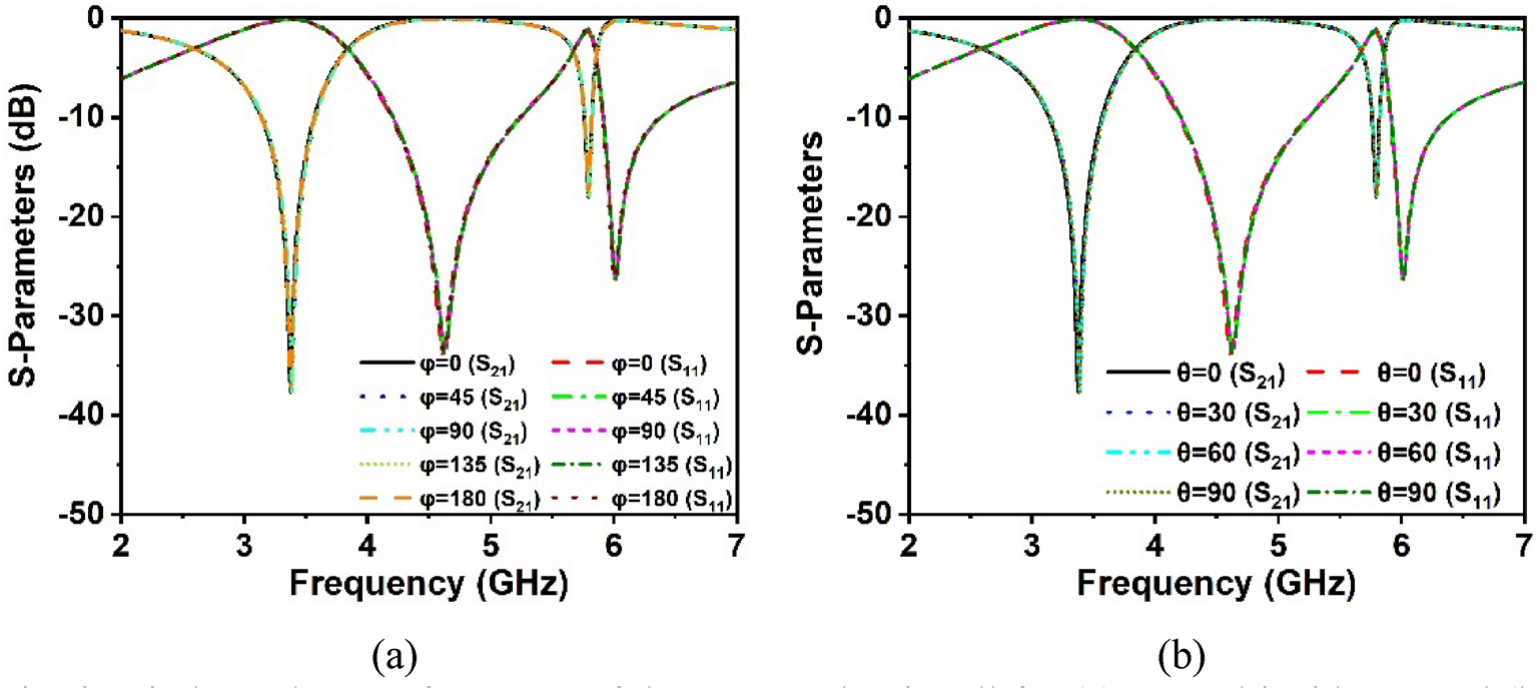
Polarization-independent performance of the proposed unit cell for (**a**) normal incidence and (**b**) oblique incidence.

**Figure 10:**
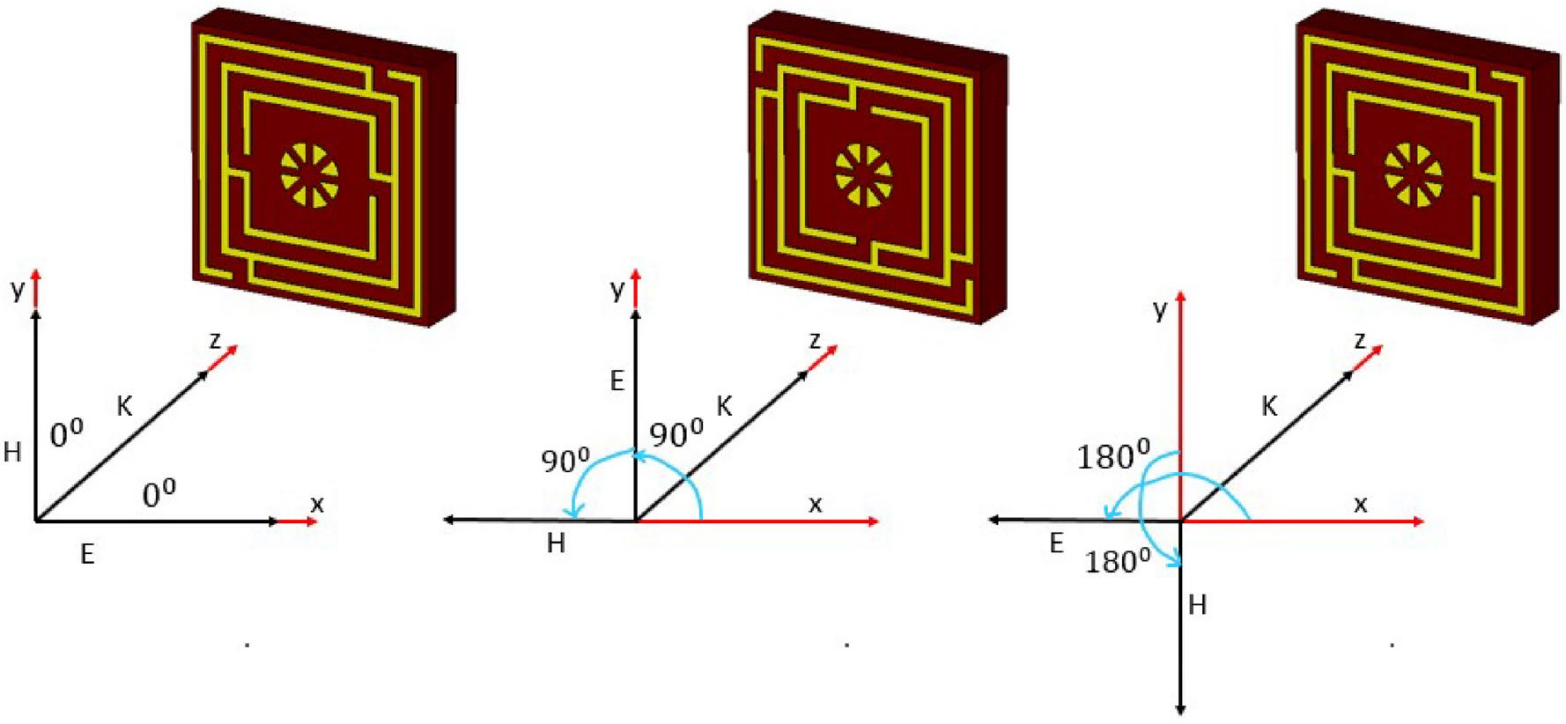
Polarization insensitivity at various angles of incidence (CST STUDIO SUITE 2019, https://www.3ds.com/products-services/simulia/products/cst-studio-suite)^39^.

The polarization insensitivity of the suggested MM structure has been examined using incident angle (θ) of 0°–90° and polarization angle (φ) of 0°–180°, as demonstrated in Fig. 9a,b. The wave vector of the propagation k is in the z-direction, with the E and H vectors in the x and y axes, respectively, as shown in Fig. 10. The proposed MM unit cell exhibits polarization insensitivity at all incidence angles, as noticed in Fig. 9a,b. The MM unit cell shows comparable resonances under all instances, including the oblique and normal incidence of electromagnetic waves. Thus, the proposed structure is independent of all polarizing angles owing to its unique design geometry. As the unit cell response is the same for all angles of incidence, the proposed MM structure might be a potential choice for 5G applications.

### Parametric study

**Figure 11:**
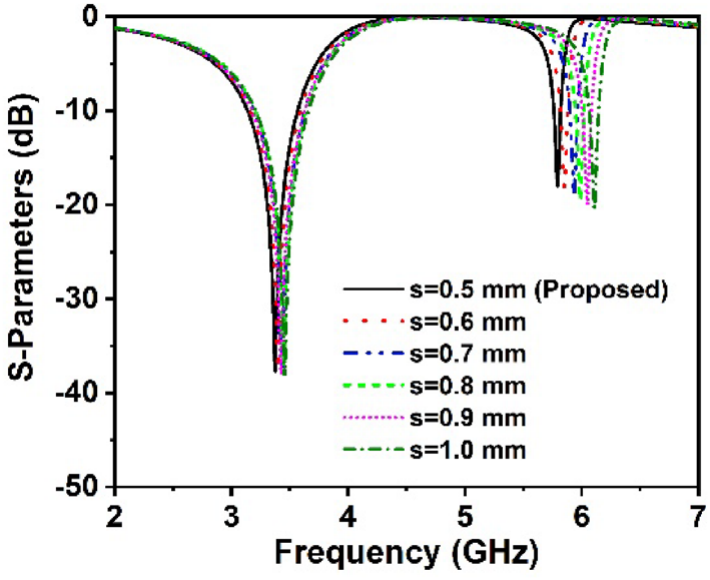
Comparison representations for various split gaps of the proposed resonator.

This study examines the proposed unit cell's performance in the desired 5G band, as well as the effects of changing the split gap of the ring. The split gap provides a capacitive effect, while the square ring acts as an inductor, resulting in an LC circuit with a resonance frequency that is dependent on the L and C values. As a result, the resonances of the MM unit cell are likely to be controlled by varied split gaps. The width of split gaps is considered in this analysis to be between 0.5 and 1.0 mm. Figure 11 shows the unit cell transmission coefficients for the various split gaps ‘*s*’. The resonances of the proposed unit cell change dramatically in the higher frequency range when the resonator split gap width widens, as seen in Fig. 11. The lower resonance transmits frequencies between 3.3 and 3.5 GHz, whereas the higher resonance transmits frequencies between 5.8 and 6.1 GHz, encompassing the entire sub-6 GHz 5G frequency band. The significant frequency shift occurs in the higher frequency resonance in contrast to the lower frequency resonance once the width of the split gaps is increased, as illustrated in Fig. 11. These studies indicate that the suggested unit cell is capable of working throughout the full sub-6 GHz 5G frequency spectrum, making it a viable contender for future wireless communication.

## Surface current, magnetic field and electric field analysis

Different forces and fields exist because of the generated charge, which influences metamaterial electromagnetic characteristics. Maxwell's curl equations^42^ can be used to represent the produced electric and magnetic fields. The equations of Maxwell are represented by the differential expressions below.

7 $$\nabla \times {\varvec{E}} = - \frac{{\partial {\varvec{B}}}}{\partial t}$$

8 $$\nabla \times {\varvec{H}} = {\varvec{J}} + \frac{{\partial {\varvec{D}}}}{\partial t}$$

where,

9 $$\nabla = \left[ {\frac{\partial }{\partial x},\frac{\partial }{\partial y},\frac{\partial }{\partial z}} \right]$$

Here, ***B***, ***D***, ***E***, and ***H*** stand for magnetic flux density, electric flux density, induced electric field density, and induced magnetic field density, respectively, while ***J*** stands for electric current density. Equation (7) expresses Faraday's equation of electromagnetic induction, whereas expression (8) is a modified version of Ampere's law that includes displacement current, $\frac{{\partial {\varvec{D}}}}{\partial t}$. To evaluate the electromagnetic fields' relationships with metamaterial characteristics, the following equations must also be addressed.

10 $${\varvec{D}} \left( t \right) = \varepsilon \left( t \right) \times {\varvec{E}}\left( t \right)$$

11 $${\varvec{B}} \left( t \right) = \mu \left( t \right) \times {\varvec{H}}\left( t \right)$$

**Figure 12:**
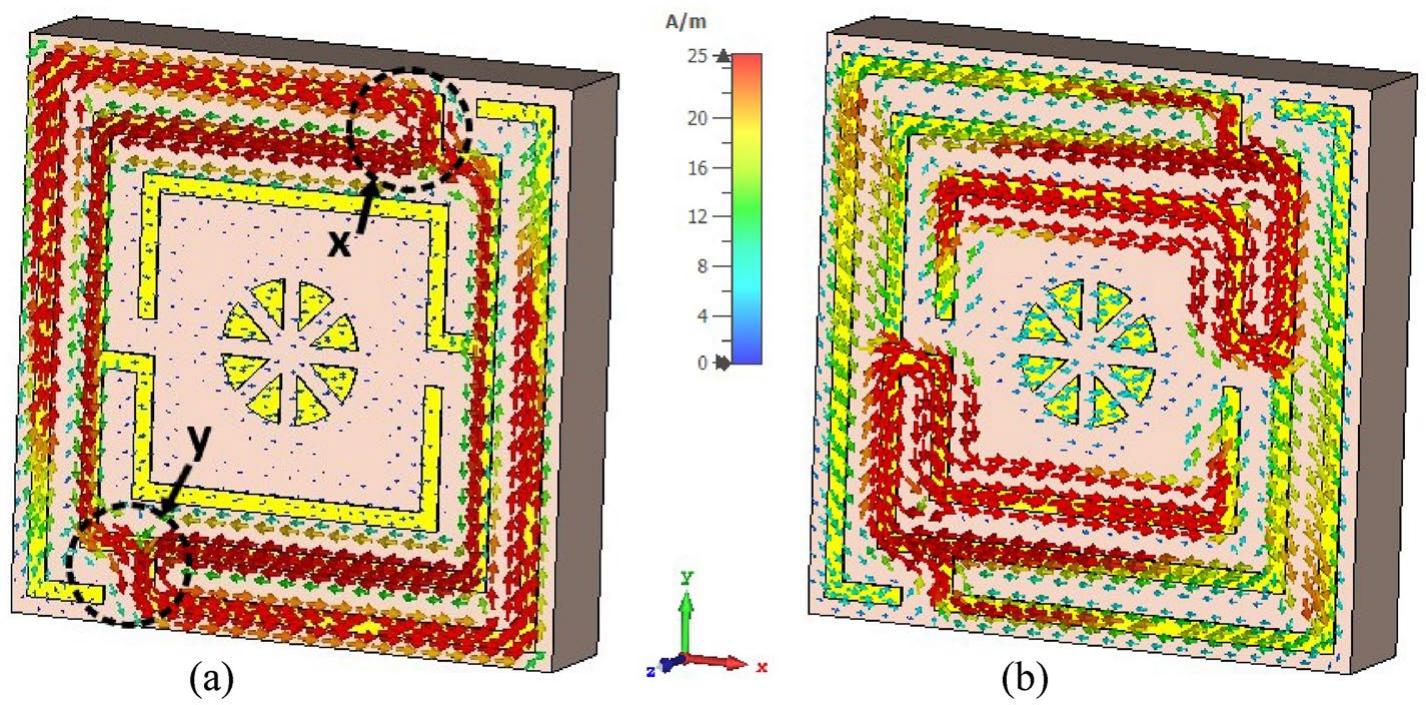
Surface current distribution for the presented MM structure at (**a**) 3.37 GHz and (**b**) 5.8 GHz (CST STUDIO SUITE 2019, https://www.3ds.com/products-services/simulia/products/cst-studio-suite)^39^.

**Figure 13:**
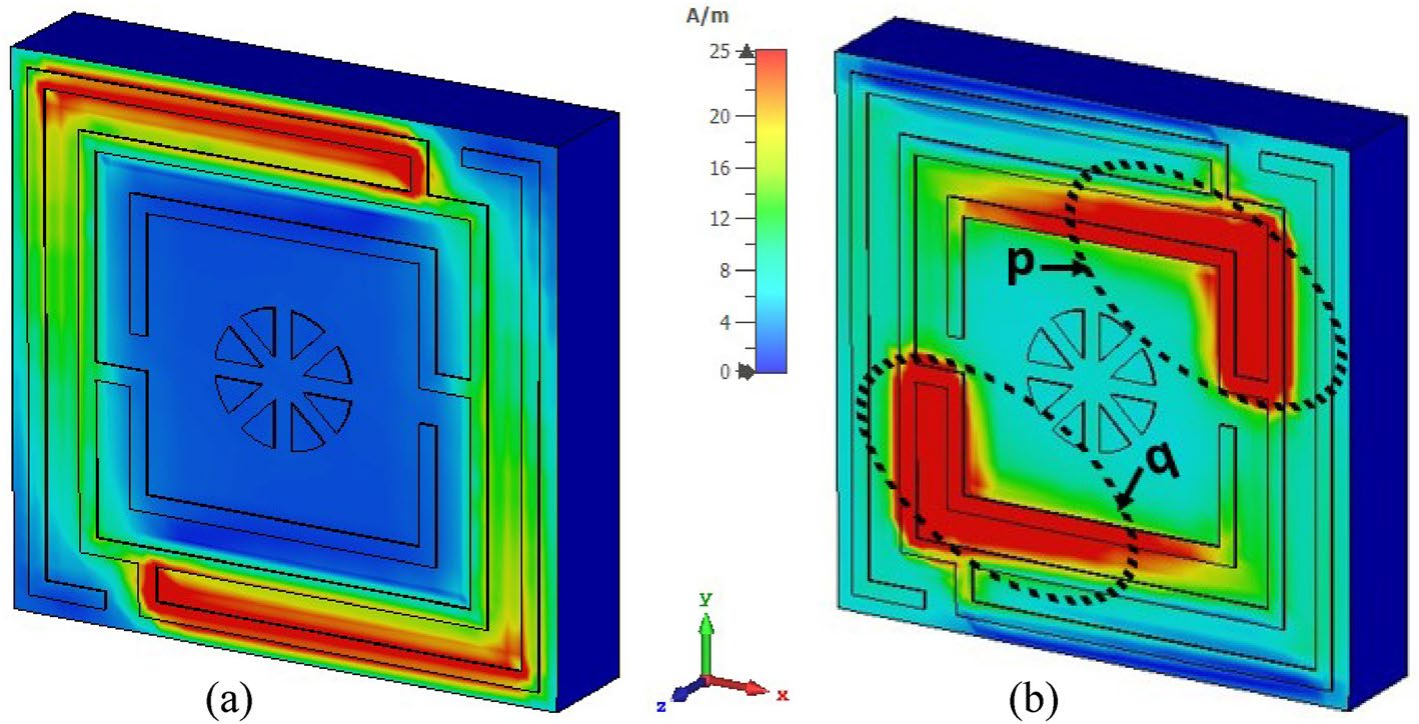
H-field distribution for the presented MM structure at (**a**) 3.37 GHz and (**b**) 5.8 GHz (CST STUDIO SUITE 2019, https://www.3ds.com/products-services/simulia/products/cst-studio-suite)^39^.

**Figure 14:**
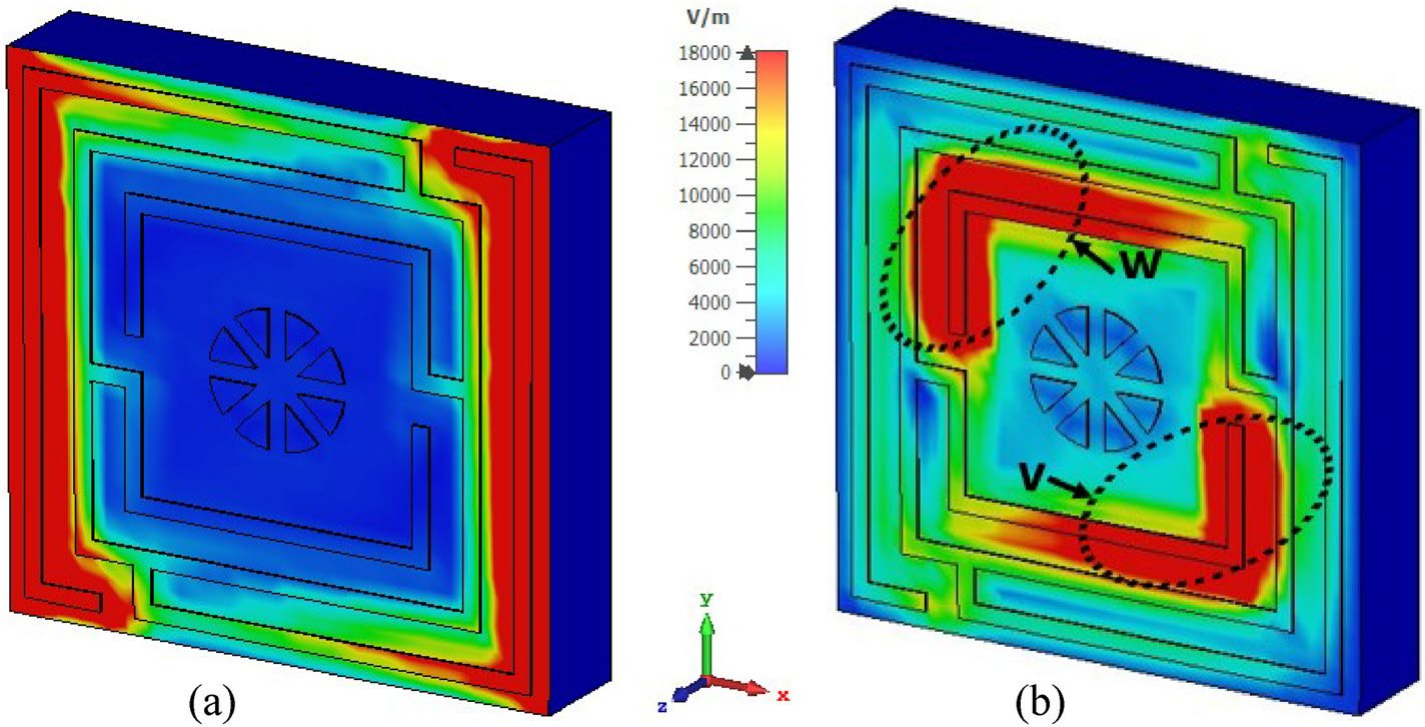
E-field distribution for the presented MM structure at (**a**) 3.37 GHz and (**b**) 5.8 GHz (CST STUDIO SUITE 2019, https://www.3ds.com/products-services/simulia/products/cst-studio-suite)^39^.

Surface current, magnetic, and electric field distributions of the proposed unit cell are presented in Figs. 12, 13, and 14, respectively, to study the association among surface current, magnetic field and electric field corresponding to the above given equations. A magnetic field source is an electric charge that varies over time. In turn, this changing magnetic field produces a varying electric field. This interconnection of electric current, electric fields, and magnetism impacts resonance occurrences. Figure 12 illustrates the surface current pattern of the produced MM at 3.37 GHz and 5.8 GHz, respectively, where the diagonal corner dominates the current density. As illustrated in Fig. 12a, the opposite diagonal corner of the top and middle rings exhibits the most substantial current, whilst the central circular pattern and bottom ring exhibit the lowest current. The current direction in these top two rings is antiparallel, therefore, the mutual coupling effect is cancelled out, as shown in Fig. 13a. It is also observed that the metal connecting strip generates a path to add and split current between the upper and middle rings, as shown in Fig. 12a by the circles x and y. In the distribution of magnetic fields, it is found that the magnetic field strength is more significant in areas with a high electric current density as per Eq. (8). As the distance from the metal ring rises, H field intensity falls (shown in Fig. 13a), satisfying the equation ${\varvec{B}} = \mu I/2\pi r$, where μ is the permeability constant, and *r* is the distance from the metal wire. The magnetic field is weakest in the middle circular geometric pattern and bottom ring illustrated in Fig. 13a. According to Maxwell's Eq. (7), the electric and magnetic fields display antagonistic excitation. The strong electric field is observed in the vertical arms of the outer ring, as shown in Fig. 14a, which satisfy the Eq. (7). Furthermore, the electric field is more significant at the split points due to the capacitance effect and at a location where there are high magnetic field fluctuations, as seen in Fig. 13a. Now, an investigation of Fig. 12b for 5.8 GHz shows a less dense current found in the central geometric pattern of the unit cell, whereas current through the vertical arms of the outer ring is nearly zero. Notably, a significant electric current persists at the diagonal corner of the middle and lower rings, which is the reverse of what occurs at the 3.37 GHz resonance. As with 3.37 GHz, antiparallel current direction appears at the horizontal arms of the bottom and middle rings, which cancels out the mutual coupling effect, as seen in Fig. 13b. It is observed that a strong magnetic field is experienced in the region p and q (shown in Fig. 13b) owing to the high-density surface current. The subsequent electric field distribution at 5.8 GHz reveals that a significant electric field is indeed in the area w, v due to the changing magnetic field at the corresponding position, as seen in Fig. 14b. However, the electric field is almost negligible in the inner circular pattern due to the absence of fluctuation in the magnetic field.

## Equivalent circuit of the proposed MM unit cell

The suggested unit cell resonator geometry encloses a center circular geometric pattern within the three square split coupled rings, demonstrating capacitive and inductive effects. The inductance is induced by the metal strips, whereas split gaps contribute to the capacitance of the structure, which controls the resonance frequency of the resonator. By combining the split and the electric field, it is possible to produce electric resonance. Conversely, when a resonating structure interacts with an electromagnetic wave, magnetic resonance is created by the combination of metal loops and magnetic field response. Thus, the whole structure resembles an LC circuit that exhibits resonance, with resonance frequency $\left( f \right)$ having the following relation^30^:

12 $$f = \frac{1}{{2\pi \sqrt {LC} }}$$

where *L* and *C* are the inductance and capacitance of the resonator. The capacitance, *C*, owing to the split gap, can be determined by using the relation as expressed in Eq. (13):

13 $$C = \varepsilon_{0} \varepsilon_{r} \frac{A}{d}\left( F \right)$$

here, $\varepsilon_{0}$ and $\varepsilon_{r}$ denote the free space permittivity and relative permittivity, respectively, whereas *A* and *d* indicate the metal strip area and split distance, respectively. The inductance of the resonator ring can be computed by applying the transmission line principle equation^22,43^:

14 $$L\left( {nH} \right) = 2 \times 10^{ - 4} l\left[ {\ln \left( {\frac{l}{w + t}} \right) + 1.193 + 0.02235\left( {\frac{w + t}{l}} \right)} \right]K_{g}$$

**Figure 15:**
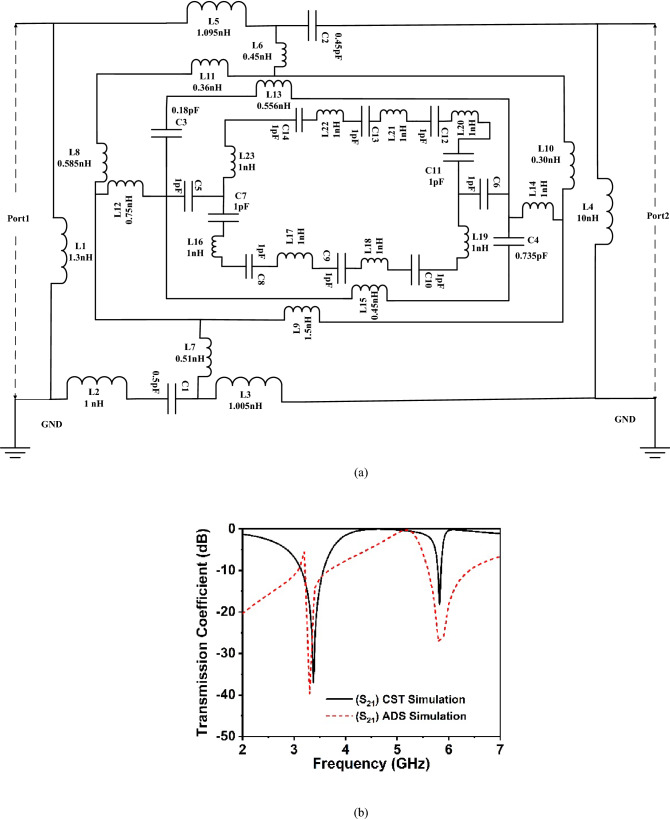
(**a**) Equivalent circuit (EC) model of the proposed CRECGR unit cell and (**b**) comparison of S_21_ for CST and equivalent circuit.

Here, the correction factor,$K_{g} = 0.57 - 0.145 {\text{ln}}\frac{{w^{\prime}}}{{h^{\prime}}}$, in which $w^{\prime}$ and $h^{\prime}$ are the substrate width and thickness, respectively. Moreover, $t, l$ and $w$ denote the thickness, length and width of microstrip lines, respectively. The equivalent circuit of the proposed metamaterial unit cell is drawn in Fig. 15a, which considers the inductive and capacitive effect of each ring as well as the split gaps. In Fig. 15a, L1, L2, L3 L4 and L5 represent the inductive effects of the outer ring, whereas *C1* and *C2* represent the capacitance associated with the split gaps in this ring. Inductances *L6* and *L7* are used to connect the first and second split rings. Besides inductances *L8*, *L9 L10,* and *L11* are corresponding inductances of different arms in the second inner ring, while *L12*, and *L14* are the inductance of the metallic strip for the second and third inner ring connector. Inductances of *L15,* and *L13* represent the inductive effects of the third ring whereas *C3* and *C4* represent the capacitance associated with the split gaps in the third split ring. Finally, *C5* and *C6* represent the capacitance between the split gap of the third ring and parasite element. There are eight parasite element and eight parasite gap which is represented by eight inductance (*L16-L23*) and eight capacitances (*C7-C14*).

The equivalent circuit is modelled in an advanced design system (ADS), and the effect of the various inductances and capacitances are investigated by tuning these component values. The numerical value of these circuit components is determined by tuning their values in ADS so that the equivalent circuit provides two similar resonances of S_21_ at 3.30 GHz and 5.80 GHz. The inductor-capacitor pair *L11* & *C3* controls the lower resonance frequency at 3.3 GHz. Changing the values of these components causes a significant deviation in frequency for the resonance at 3.30 GHz. A noticeable effect of *L12-C2* is experienced on the higher resonance occurred at 5.80 GHz. Effect of the inner ring inductances is observed on the second resonance at 5.80 GHz. The transmission coefficient response for circuit simulation in ADS is compared with the similar response of the proposed metamaterial at CST simulation in Fig. 15b. The S_21_ figure from the ADS circuit simulation is much like the CST S_21_ plot, as seen in Fig. 15b. A slight bandwidth mismatch is observed for the resonance at the lower frequency. This is because, in CST simulations, the metamaterial's inductive action is distributed uniformly throughout the resonator. Moreover, it is suffered by the coplanar capacitances existing between two adjacent metallic sides of the rings. However, in circuit simulation, the effect of the coplanar capacitor is neglected. Furthermore, circuit simulation treats components as lumped, resulting in a bandwidth deviation from the CST simulation result.

## Fabrication and experiment

**Figure 16:**
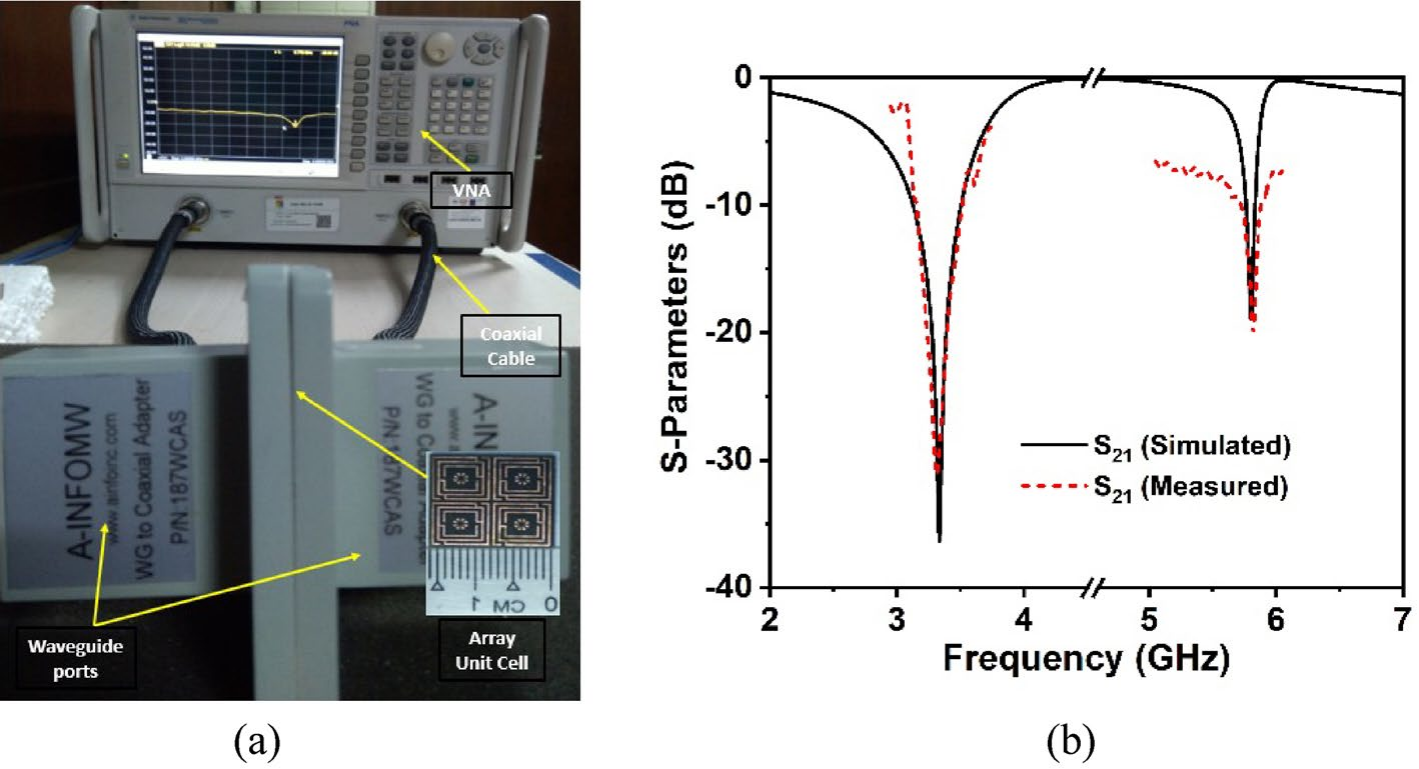
(**a**) Experimental arrangement with assembly parts and (**b**) comparison of measured and CST simulated S_21_ responses for a developed 2 × 2 array structure.

The prototype of the proposed 2 × 2 array structure is fabricated to confirm the CST simulation findings by measuring the practical response depicted in Fig. 16a,b. Figure 16a shows the measuring equipment setup for the developed metamaterial structure at UKM's microwave laboratory, which includes two different waveguide ports and a two-port PNA network analyzer (model: Agilent N5227A). The manufactured array prototype is sandwiched between the two waveguide ports that are connected to the PNA network analyzer through the probe. The measured finding of the array prototype is presented in Fig. 16b alongside CST simulation results. According to the observed results, the measured resonances are very closely matched to those obtained by simulation. In addition, the measured -10 dB S_21_ bandwidth expands from 3.2 to 3.6 and 5.7–5.9 GHz, respectively covering the sub-6 GHz 5G spectrum, which matches the CST simulated values. Despite a bit of variation in the frequency response, the simulated and experimental findings are fairly close. As illustrated in Fig. 16b, the peak amplitudes of the measured resonances deviated somewhat from the simulated ones. Fabrication flaws, a small gap between the edges of the fabricated prototype and waveguide ports, cable loss, mutual coupling effects between two ports or two array components, and experimental open-air conditions are responsible for these discrepancies between observed and simulated findings. Nevertheless, beyond these constraints, the MM array exhibits excellent performance with a high correlation between simulation and experiment, making it perfect for 5G wireless communication applications.

## Absorption properties of the proposed MM structure

**Figure 17:**
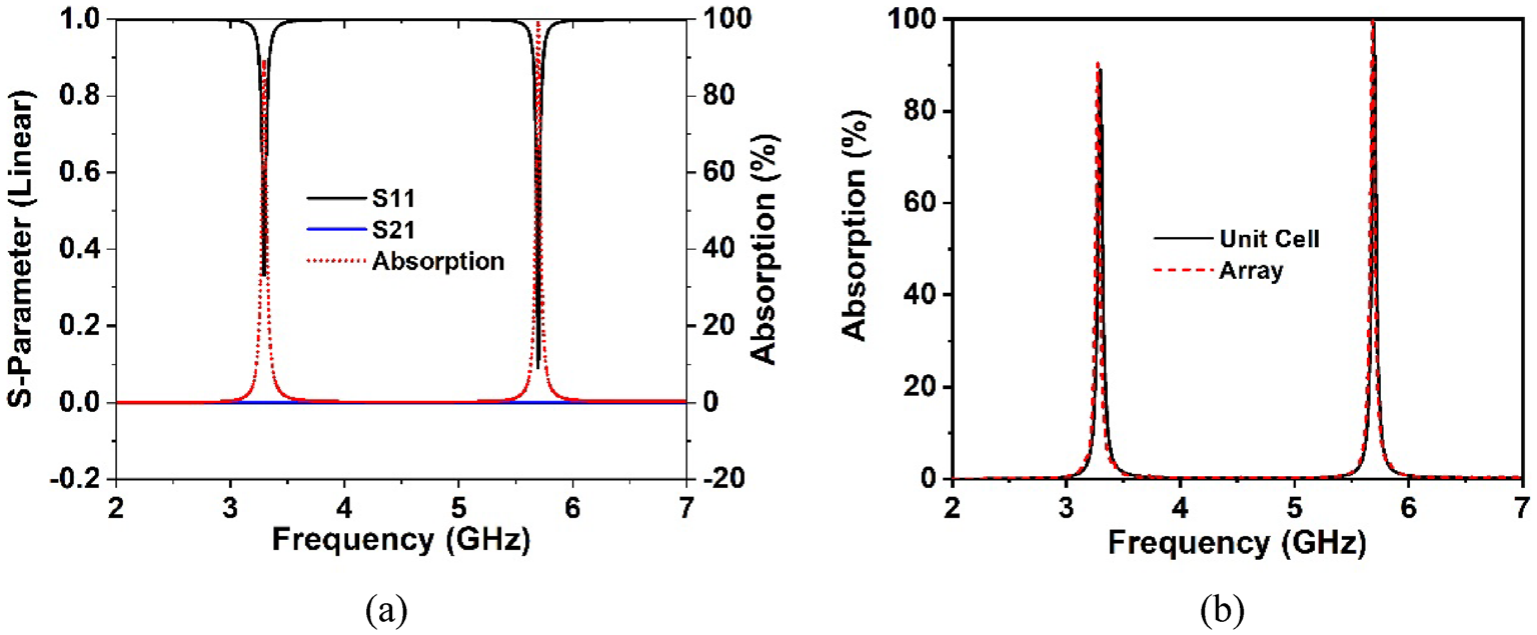
(**a**) Absorption and scattering parameters of the suggested unit cell and (**b**) unit cell and array absorption performance.

When a copper backplane is embedded into the proposed metamaterial unit cell, it serves as an absorber. The metamaterial absorber absorbs electromagnetic energy by introducing magnetic and electric resonance with zero transmission waves. The reflection, transmission and absorption coefficient performance spectra are illustrated in Fig. 17a. Due to the presence of copper in the backplane, it is noticed that the incident wave transmission is zero. When backside copper is incorporated into the metamaterial, a current is induced in the back copper, which forms a magnetic field that prevents transmission by interfering with the transmitted wave. Hence, almost all transmitted EM waves are absorbed since the transmitted wave penetration depth is less than the back copper thickness (0.035 mm). At 5.7 GHz resonance, the unit cell displays absorption close to unity, while at 3.3 GHz resonance, it exhibits absorption exceeding 91 percent. Thus, absorption is maximum where the reflection spectrum is least and vice versa, as shown in Fig. 17a. Using the following general formula, the absorption has been computed from the S_11_ and S_21_ parameters:

15 $$A = 1 - \left| {S_{11} } \right|^{2} - \left| {S_{21} } \right|^{2}$$

**Figure 18:**
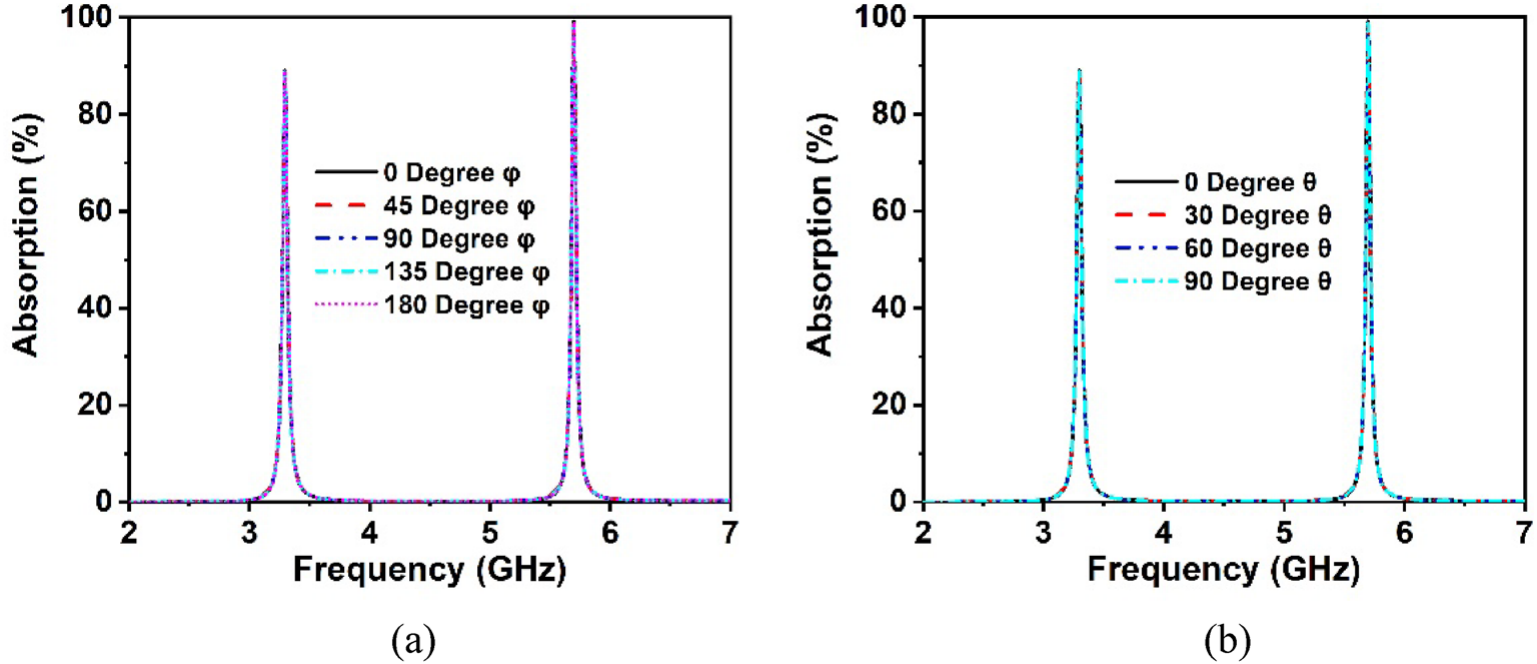
Absorption performance of the proposed MM structure (array) for (**a**) normal angle of incidence and (**b**) oblique incidence.

**Figure 19:**
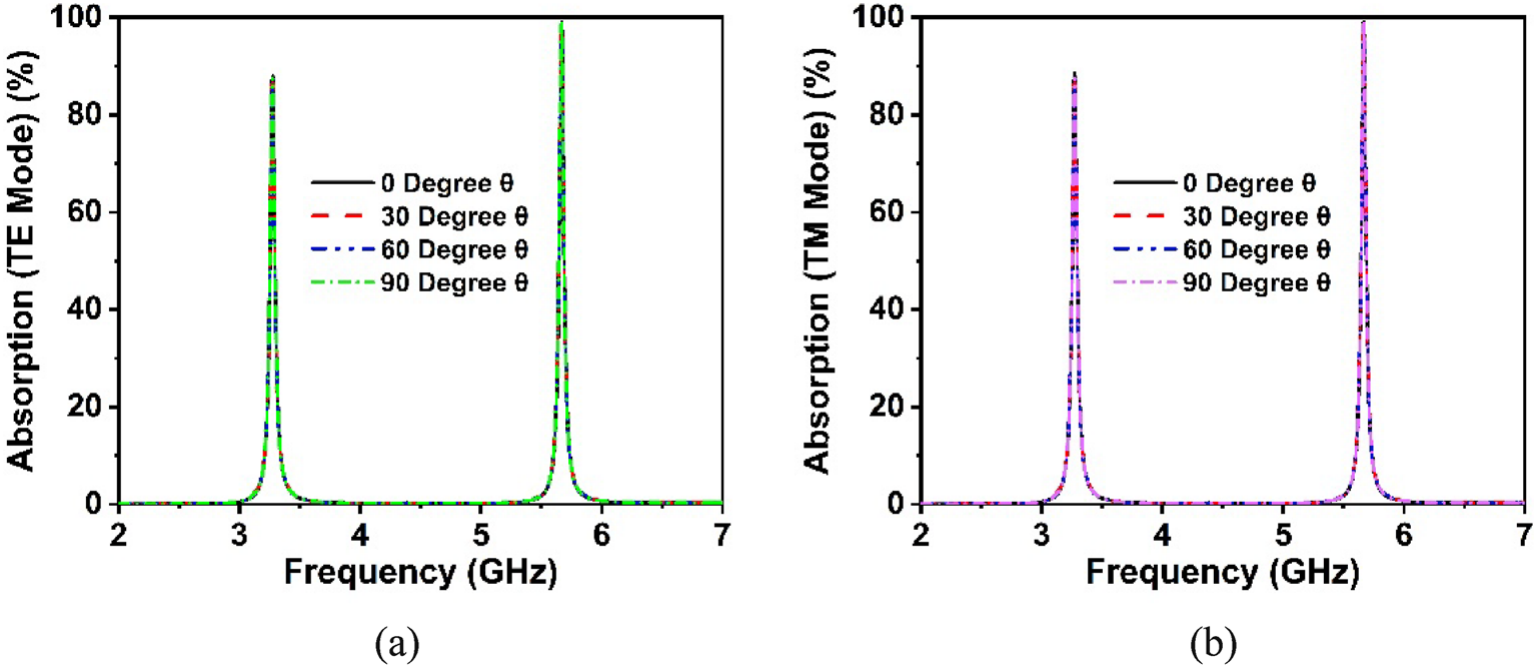
Absorption characteristics for various angles of oblique incidence in (**a**) TE and (**b**) TM modes of operation.

Figure 17b shows the comparative analysis of the absorption performance of the proposed unit cell and its array arrangement. The absorption values of the unit cell and array are 91% and 91.2% at 3.3 GHz, respectively, and 99.2% and 99.9% at 5.7 GHz, respectively. It is worth noting that the absorption of the unit cell and its array is nearly identical, even though the array performs better at both resonances than the unit cell. Furthermore, the simulation is performed in terms of normal and oblique EM wave incidence for a 2 × 2 unit cell array, as exhibited in Fig. 18a,b. The proposed absorber offers uniform absorption for all angles of incidence, exhibiting angle insensitivity because of its axis symmetrical structure. Thus, the suggested metamaterial absorbs a maximum of 99.9% of energy at all polarizing angles. The effect of different oblique incidence angles on the absorption performance of the proposed MM for TE (Transverse Electric) and TM (Transverse Magnetic) polarized electromagnetic (EM) waves have been explored, and the simulated results are shown in Fig. 19a,b. It is worth noting that the suggested structure ensures near-unity absorption at higher frequencies for obliquely incident EM waves in both the TE and TM modes. Moreover, the absorption in TE and TM mode is unaffected by oblique incidence angles. Figure 19a,b demonstrates the almost identical absorption characteristics of TE and TM mode of operation, which covers the 5G sub-6 GHz frequency spectrum. It is noticeable to mention that the proposed MM absorber is insensitive to oblique incidence angles of up to 90° for EM waves in the TE and TM modes. Moreover, the bandwidth of the absorption spectra is quite narrow, indicating an excellent quality factor of 110 and 285 for 3.3 GHz and 5.7 GHz, respectively, as determined by the equation, $Q={f}_{0}/HMBW$, where ${f}_{0}$ is the absorption frequency and HMBW is the half-power maximum bandwidth^27^. All of these characteristics imply that the suggested MM structure with copper backplane has outstanding absorption properties and is suitable for sensing and energy harvesting in the 5G spectrum^44^.

**Figure 20:**
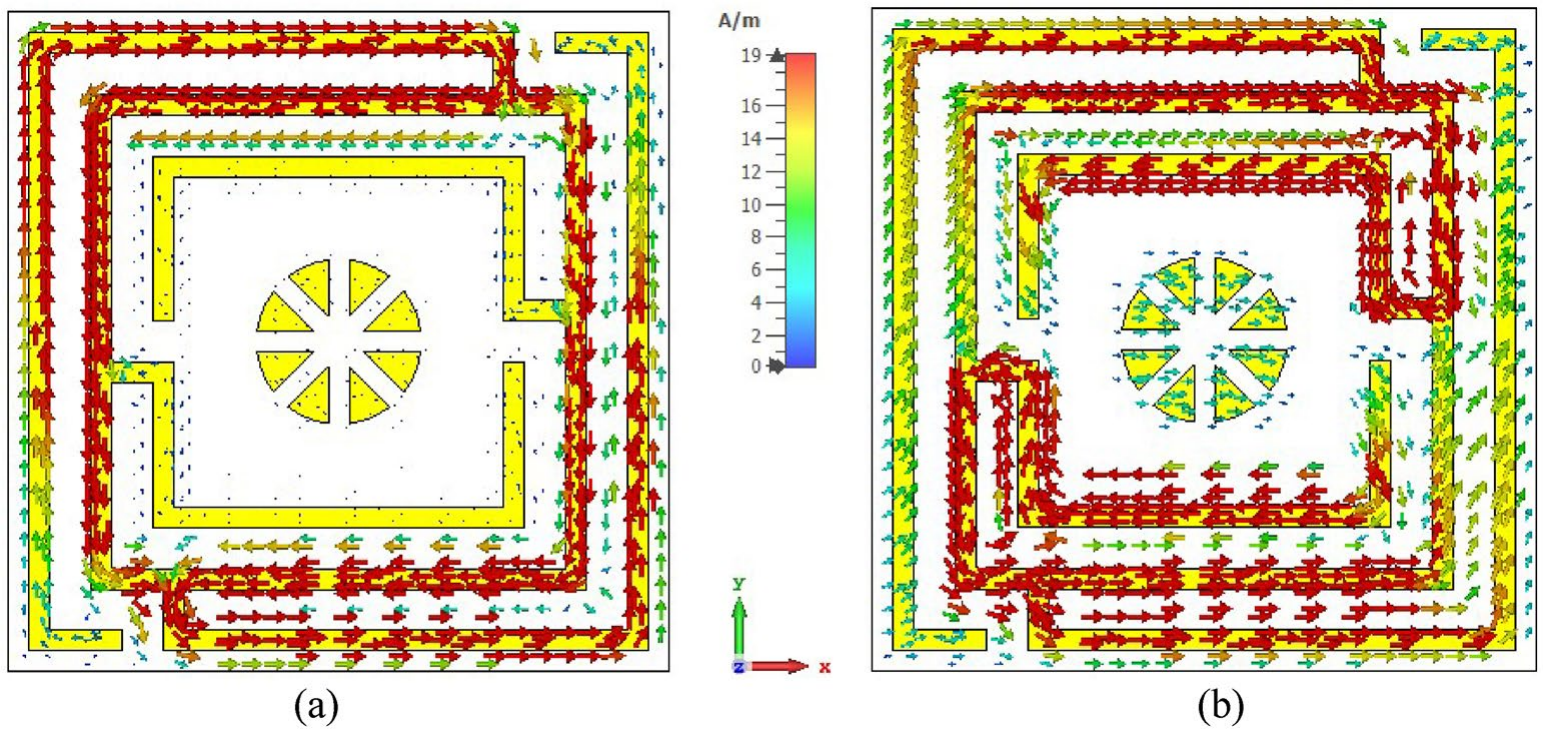
Surface current circulation in absorber resonator patches at (**a**) 3.3 GHz and (**b**) 5.7 GHz (CST STUDIO SUITE 2019, https://www.3ds.com/products-services/simulia/products/cst-studio-suite)^39^.

**Figure 21:**
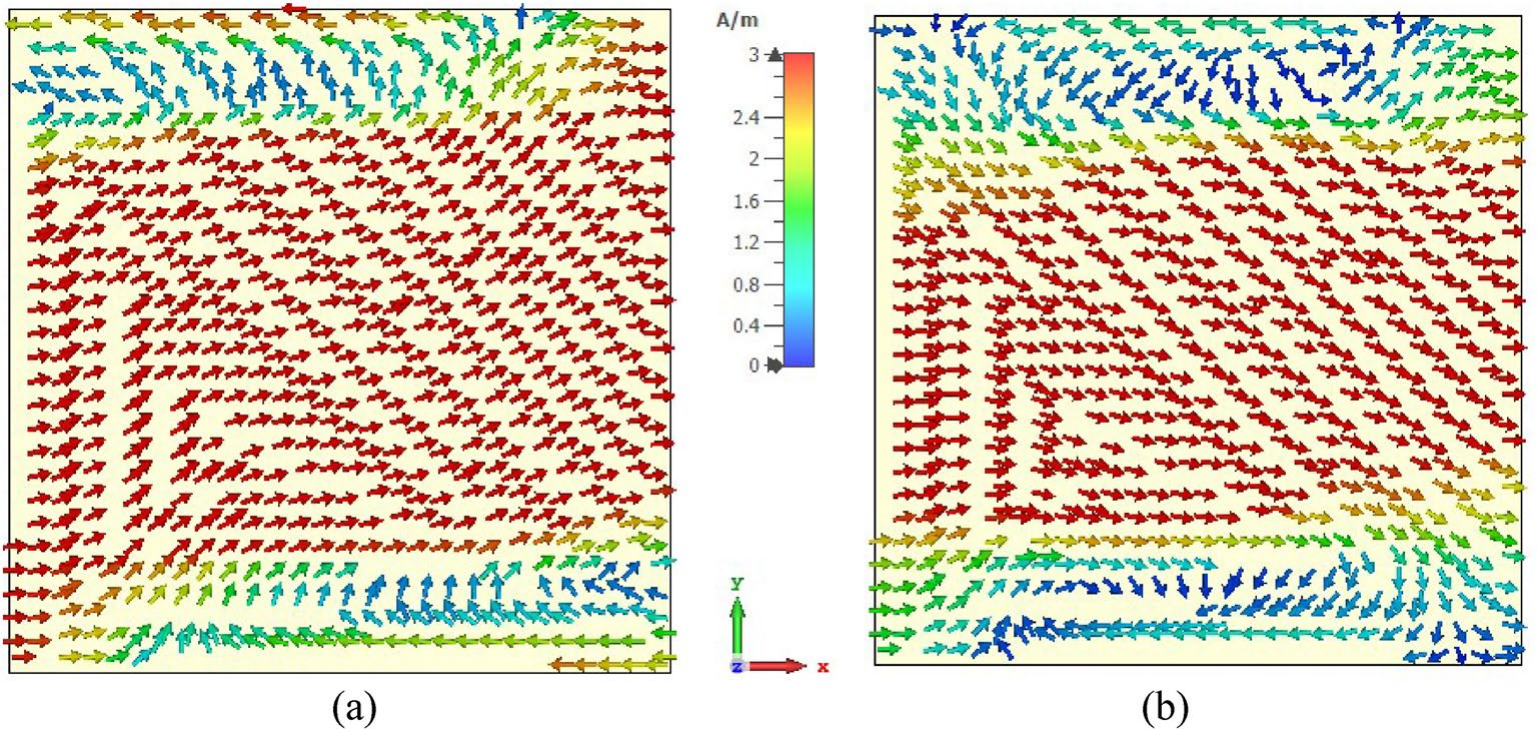
Copper backplane surface current distribution at (**a**) 3.3 GHz and (**b**) 5.7 GHz for absorber resonator (CST STUDIO SUITE 2019, https://www.3ds.com/products-services/simulia/products/cst-studio-suite)^39^.

The top and back surface currents pattern of the suggested unit cell absorber has been investigated to realize the absorption process and resonance characteristics, as shown in Figs. 20 and 21. The mechanics of absorptance is that antiparallel current is generated on the front and back sides of the absorber, resulting in a current loop and magnetic resonance, which is liable for absorption^45^. The surface current distribution of the absorber front side (Fig. 20a) for 3.3 GHz reveals a significant antiparallel current in the diagonal corner of the top and middle square rings, with the bulk of current passing on the middle ring. In the copper backplane depicted in Fig. 21a, an antiparallel current is investigated at the upper and lower ends of the top and middle rings. Magnetic resonance is generated by the antiparallel current, which leads to the absorption of almost 91% at 3.3 GHz. On the other hand, with the 5.7 GHz resonance (shown in Fig. 20b), the maximum current is found on the top and lower sides of the three-square rings, whereas the parallel current is observed in the upper two rings. In the copper backplane, the antiparallel current is noticed at the exact location as on the front side displayed in Fig. 21b. These three antiparallel currents offer the maximum absorption of 99.2% at 5.7 GHz. It is noticeable that the absorption observed at 3.3 GHz and 5.7 GHz frequencies, which is very close to the resonances of the proposed resonator.

## Experimental output of the CRECGR MM absorber

**Figure 22:**
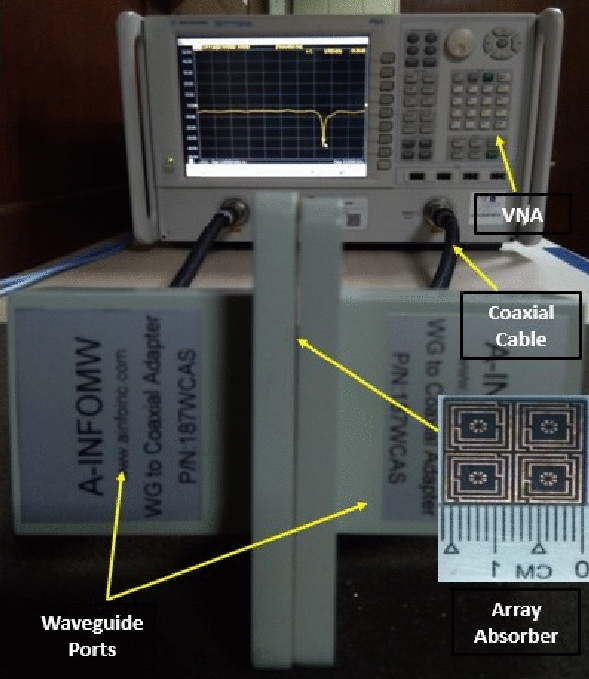
CRECGR metamaterial absorber experimental setup with assembly components.

**Figure 23:**
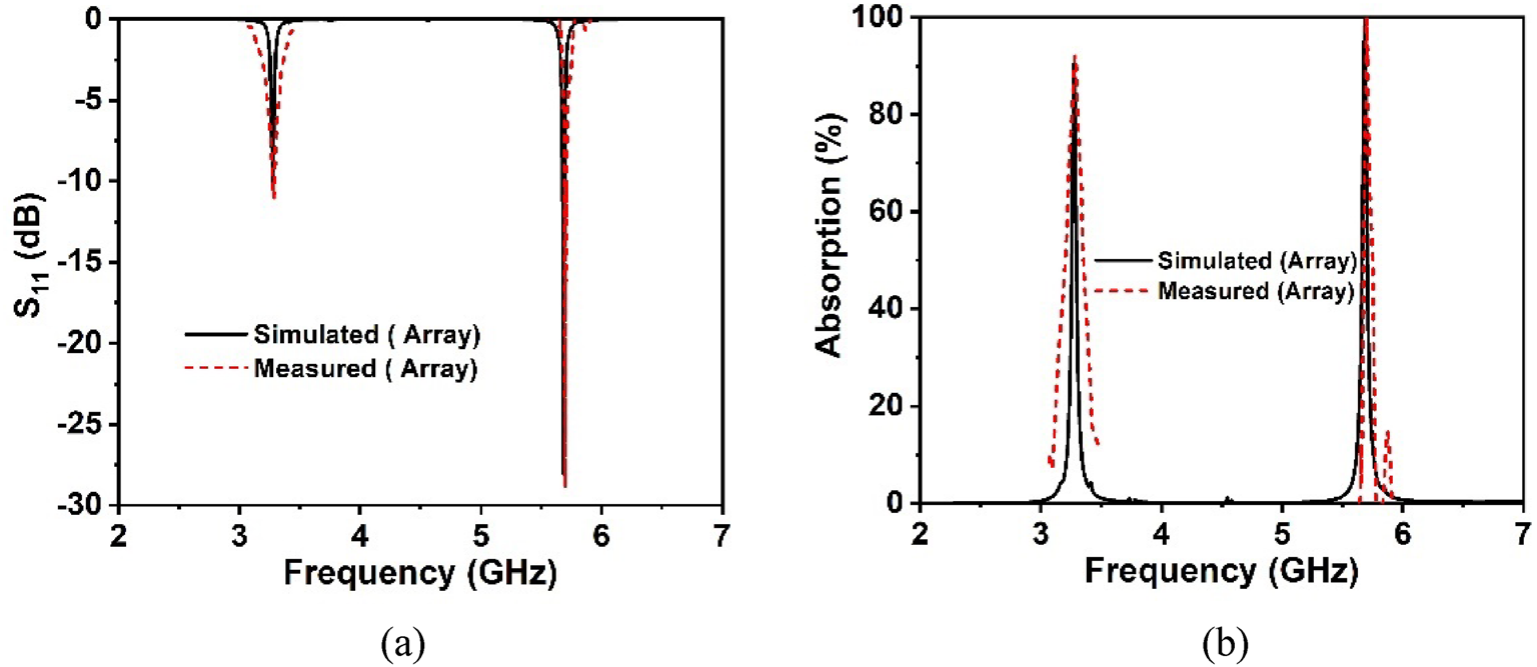
Measured and CST simulated results (**a**) S_11_ response in dB and (**b**) absorption for a fabricated 2 × 2 array of the absorber.

The 2 × 2 array of the unit cell absorber is fabricated for the purpose of performing measurement and confirming performance to the simulation findings. The photograph of the fabricated prototype as well as the experimental procedure with a PNA series vector network analyzer and two waveguides are presented in Fig. 22. The experimental reflection coefficient values have been collected from the measurement setup, and the absorption performance extracted from the measured data using the formula provided in Eq. (15), as illustrated in Fig. 23a,b, respectively. The suggested unit cell with a copper backplane shows excellent absorption at the resonances depicted in Fig. 23b, with maximum absorption of 99.9% at 5.7 GHz. It can address that the measured and simulated findings are in excellent agreement with slight deviation, as shown in Fig. 23a,b. This divergence happened as a result of coaxial connector loss, edges mismatch between fabricated prototype and waveguide ports, a coupling effect between waveguides and the open-air testing environment, which contributed to erroneous absorption and bandwidth displacement. Notwithstanding these influences, the 2 × 2 array absorber achieves outstanding absorptions at the desired resonances, making it suitable for 5G applications.

## Sensing applications

**Figure 24:**
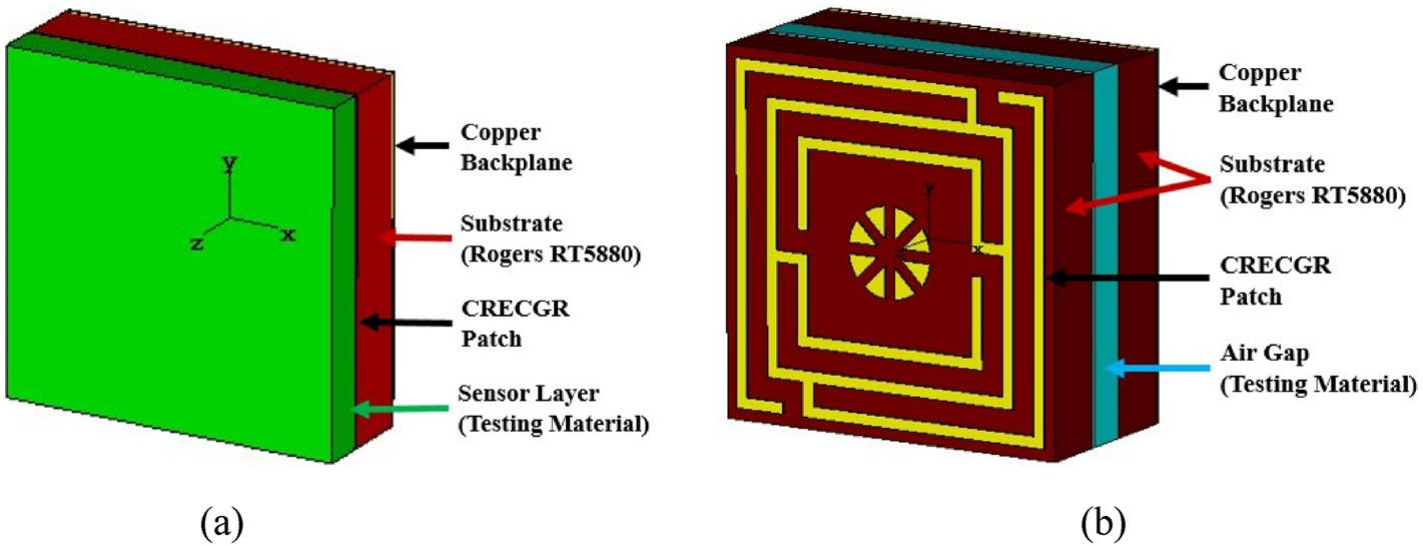
(**a**) Model for sensing solid materials and (b) Liquid (Edible oil) sensing model. (CST STUDIO SUITE 2019, https://www.3ds.com/products-services/simulia/products/cst-studio-suite)^39^.

**Figure 25:**
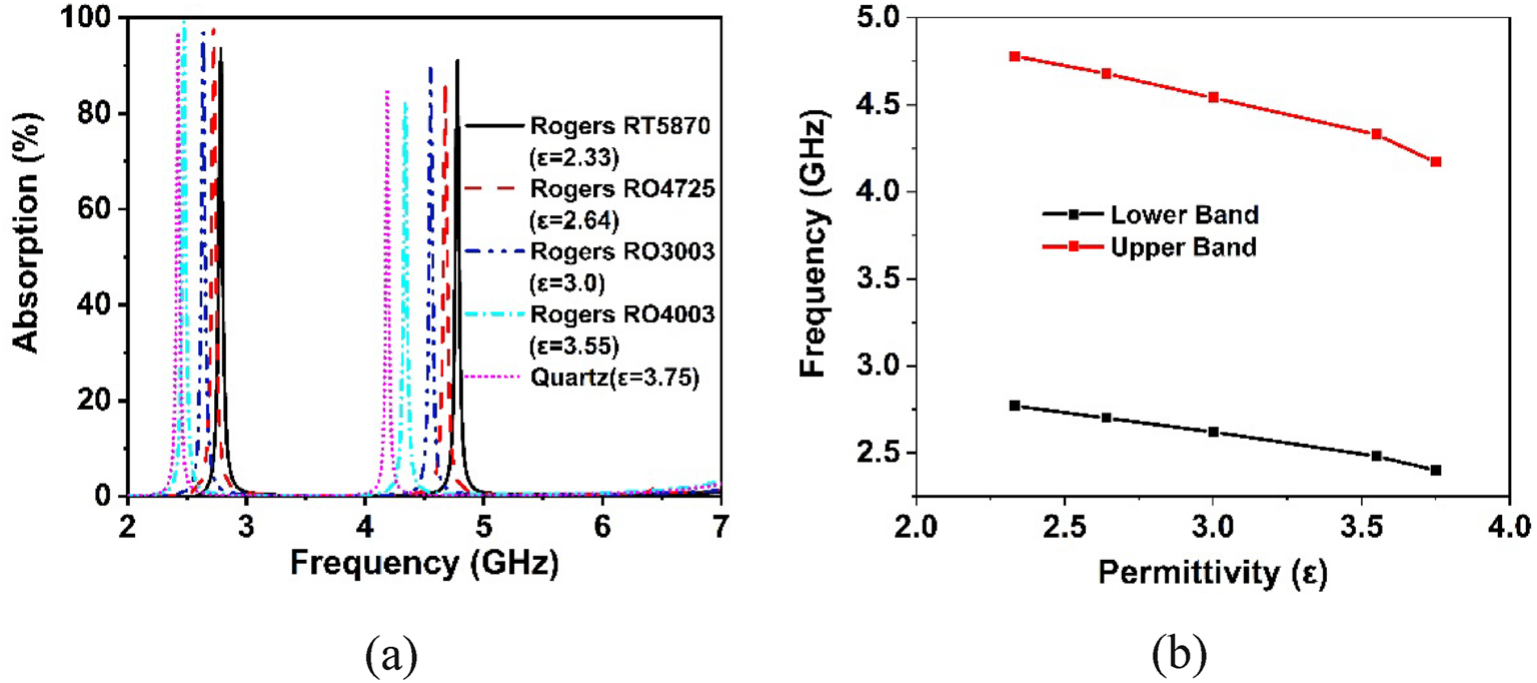
(**a**) Absorption responses of various sensing materials and (**b**) Plot of dielectric constant against frequency at the point of maximum absorption.

**Figure 26:**
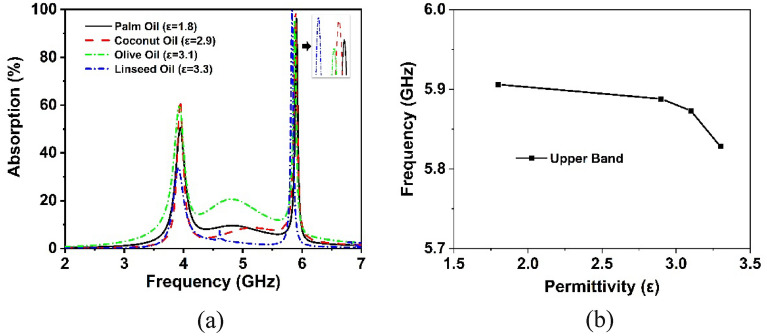
(**a**) Absorption properties of various liquid (Edible oil) materials sensing and (**b**) plot of dielectric constant against frequency at the point of maximum absorption.

In recent times, metamaterial-based sensing technology has exploded in popularity among the research community^46^. Thus, the proposed CRECGR metamaterial exhibits outstanding identical absorption qualities up to 180° incident angles with maximum absorption of 99.9%, which leads it a perfect choice for sensing applications in the 5G sub-6 GHz spectrum. Artificially engineered MMs have indeed been widely employed in a variety of applications, including antennas^47^, energy harvesting^48^, SAR minimization^22^, and a variety of microwave sensors, notably permittivity sensors^49^, pressure sensors^34^, chemical sensors^46^, and refractive index sensors^50^. The MM patch self-capacitance is greatly influenced by the effective impedance variation, which shifts the reflection and absorption peaks. Work by^43,51^ presented a permittivity sensor for microwave spectrum sensing applications based on the absorption characteristics of the MM. Figure 24a shows the sensing application setup, which includes a 1 mm thick material sensing layer on top of the developed MM absorber. In the absence of solid testing material, the air is assumed to be the sensing medium with a dielectric constant of 1, which is closely related to the permittivity of the material. Various dielectric materials with permittivity values ranging from 2.33 to 3.75 are layered on top of the suggested absorber to evaluate its sensing properties, and the obtained findings are shown in Fig. 25. It can be noticed that the permittivity of the sensing materials increased, the absorption peak moved linearly towards the lower frequency. The changing permittivity of the sensing materials has a noticeable effect on the capacitance of the MM patch, contributing to the absorption peak being shifted. Notably, high sensitivity has been demonstrated in the 5G sub-6 GHz lower and higher bands. However, the upper band absorption has been reduced slightly due to the dielectric constant of the sensing material and the modification in the size of the MM. In addition, as illustrated in Fig. 24b, a liquid substance (edible oil) sensing is also investigated. In order to study the sensing performance, various edible oil samples with different permittivity values have been inserted between substrate materials for testing purposes. The simulated absorption outcomes of the various edible oil samples are illustrated in Fig. 26. It is found that the absorption of the various edible oils shifted linearly towards the lower frequency as the increasing of the permittivity values. Absorption in the lower band is reduced owing to changes in impedance matching and sensing material, hence the higher band is recommended for edible oil sensing applications. Thus, it is feasible that the proposed metamaterial-based sensor might be used in various sensing techniques in industry, including liquid chemical and dielectric solid material sensing.

## EMR and comparative study with contemporaries

The effective medium ratio (EMR) is an influential theoretical tool used to determine the compactness of metamaterials. The developed unit cell EMR value has been determined using a pretty basic mathematical formula^21^, $EMR=\lambda /L$, where $\lambda$ implies the lowest resonance frequency wavelength and $L$ denotes the length of the developed metamaterial structure. The EMR value of the proposed MM structure is 11.13 at the resonance frequency of 3.37 GHz, indicating increase in homogeneity and smallness without any manufacturing constraints. The comparison between the recently published metamaterial structure and the suggested one is summarized in Table 3, considering the factors including unit cell size, substrate material, structure characteristics, frequency bands, absorption ability and application arena. Compared to other previously published work in Table 3, the proposed metamaterial geometry exhibits a high EMR, wide polarization independent features, and a compact footprint covering the 5G sub-6 GHz frequency spectrum with sensing applications. Furthermore, it possesses excellent absorption in the 5G sub-6 GHz frequency spectrum at almost comparable resonances. Besides that, the proposed metamaterial is based on a well-established substrate material with a low dielectric constant (DC = 2.2) and dissipation factor (DF = 0.0009 at 10 GHz), uniform electrical properties across a broad frequency range, easy cutting and patterning, and suitability for moisture environments. The authors of^21^ merely indicated absorption with a minimum of 81% without any experimental evidence and sensitivity analysis. On the other hand, the study^32^ demonstrates polarization insensitive features up to 45°, but their EMR is relatively low, and no application with experimental confirmation is demonstrated. Thus, the suggested MM structure's originality lies in its unique geometry, high EMR, polarization insensitivity up to 180°, ENG characteristics with a negative index and sensing application in the 5G sub-6 GHz frequency range. Most importantly, it displays a unique property of excellent absorption at the resonances when a complete copper backplane is employed.

**Table 3:** Compare and contrast the proposed MM structure with existing relevant design.

Ref. and Year	Unit cell shape and size	Substrate material	Characteristics	Covering Bands	EMR	Absorption Min. and Max. (%)	Maximum polarization angle insensitivity	Applications
^52^ 2016	0.118λ × 0.118λ × 0.035λ	FR-4	LHM	C	8.45	Not shown	Not confirmed	Not shown
^21^ 2020	0.118λ × 0.118λ × 0.018λ	FR4	ENG and DNG	S and X	8.4	81 and 99	Not confirmed	Microwave (proposed)
^53^ 2021	0.099λ × 0.099λ × 0.017λ	FR-4	DNG	S, X and Ku	10.07	Not shown	Not confirmed	Microwave (proposed)
^32^ 2021	0.231λ × 0.231λ × 0.044λ	Rogers RT6002	LHM	X and Ku	4.327	Not shown	45°	Communication (proposed)
Proposed	0.089λ × 0.089λ × 0.017λ	Rogers RT5880	ENG and negative Index	5G Sub-6 GHz band	11.13	91 and 99.9	180°	Absorber and sensing

## Conclusion

This scientific study introduced a polarization insensitive dual-band CRECGR metamaterial with outstanding absorptance that covers the 5G sub-6 GHz spectrum. The proposed circular geometry-based unique patch is devised on the low loss Rogers RT5880 substrate with a miniature size of 0.089λ × 0.089λ × 0.017λ. The proposed resonator demonstrates two resonances at 3.37 GHz and 5.8 GHz, which have negative permittivity, near-zero permeability, and a negative refractive index. The core circular geometric structure shifts the resonance frequency to a lower frequency, thus improving the EMR while covering the sub-6 GHz frequency spectrum used in 5G. The findings of the CST and ADS simulations are confirmed by the results of the prototyped experimental design. The surface current density, magnetic and electric field patterns are investigated to clarify the metamaterial characteristics. The developed metamaterial structure has been proven to be polarization insensitive up to 180° of incidence angle, which is unique among related metamaterials to date. Moreover, the compactness of the developed metamaterial structure is confirmed by its high EMR value of 11.13, which ensures its homogeneity features. The robust retrieval approach-based CST post-processing module has been used to explore the effective relative parameters, confirming the ENG properties. The absorption qualities of the unit cell with a full copper backplane were examined, revealing a maximum absorption of 99.9% with an excellent quality factor, confirming its potential application in 5G enabled devices for SAR reduction, energy harvesting, and sensing. In addition, the proposed absorber polarization insensitivity has been settled by angles of incidence up to 180°. Absorption in the TE and TM modes are found to be identical and insensitive to oblique angles up to 90°. The suggested unit cell sensing performance is investigated using a permittivity-based sensing model, and excellent sensing responses are observed for a variety of testing material dielectric constants. Thus, the proposed metamaterial structure might be a promising solution for sub-6 GHz applications in the 5G wireless communication.

## References

[1] Wang Z, Dong Y, Itoh T (2020). Miniaturized wideband CP antenna based on metaresonator and CRLH-TLs for 5G new radio applications. IEEE Trans. Antennas Propag..

[2] Wang Z, Li C, Wu Q, Yin Y (2020). A metasurface-based low-profile array decoupling technology to enhance isolation in MIMO antenna systems. IEEE Access.

[3] Garg P, Jain P (2019). Isolation improvement of MIMO antenna using a novel flower shaped metamaterial absorber at 5.5 GHz WiMAX band. IEEE Trans. Circuits Syst. II Express Briefs.

[4] Pandit S, Mohan A, Ray P (2020). Low-RCS low-profile four-element MIMO antenna using polarization conversion metasurface. IEEE Antennas Wirel. Propag. Lett..

[5] Sakli H, Abdelhamid C, Essid C, Sakli N (2021). Metamaterial-based antenna performance enhancement for MIMO system applications. IEEE Access.

[6] Wang M (2018). Investigation of SAR reduction using flexible antenna with metamaterial structure in wireless body area network. IEEE Trans. Antennas Propag..

[7] Hannan S, Islam MT, Faruque MRI, Rmili H (2021). Polarization-independent perfect metamaterial absorber for C, X and Ku band applications. J. Mater. Res. Technol..

[8] Amiri M, Tofigh F, Shariati N, Lipman J, Abolhasan M (2020). Wide-angle metamaterial absorber with highly insensitive absorption for TE and TM modes. Sci. Rep..

[9] Islam MT, Islam MR, Islam MT, Hoque A, Samsuzzaman M (2021). Linear regression of sensitivity for meander line parasitic resonator based on ENG metamaterial in the application of sensing. J. Market. Res..

[10] Forouzeshfard MR, Ghafari S, Vafapour Z (2021). Solute concentration sensing in two aqueous solution using an optical metamaterial sensor. J. Lumin..

[11] Tan T (2019). Renewable energy harvesting and absorbing via multi-scale metamaterial systems for Internet of things. Appl. Energy.

[12] Lu Z-Q, Zhao L, Ding H, Chen L-Q (2021). A dual-functional metamaterial for integrated vibration isolation and energy harvesting. J. Sound Vib..

[13] Bonnetier E, Nguyen H-M (2017). Superlensing using hyperbolic metamaterials: The scalar case. Journal de l’École Polytechnique Mathématiques.

[14] Alexandropoulos, G. C., Lerosey, G., Debbah, M. & Fink, M. Reconfigurable intelligent surfaces and metamaterials: The potential of wave propagation control for 6G wireless communications. arXiv preprint arXiv:2006.11136 (2020).

[15] Shlezinger N, Alexandropoulos GC, Imani MF, Eldar YC, Smith DR (2021). Dynamic metasurface antennas for 6G extreme massive MIMO communications. IEEE Wirel. Commun..

[16] Yin L, Doyhamboure J, Tian X, Li D (2018). Design and characterization of radar absorbing structure based on gradient-refractive-index metamaterials. Compos. B Eng..

[17] Zhang Y (2021). Dual band visible metamaterial absorbers based on four identical ring patches. Physica E.

[18] Misran N, Islam M, Ismail M, Yusop S (2011). Analisis pencirian parameter ketebalan dan kebertelusan substrat bagi elemen cincin segiempat sepusat bersela antena tatasusun pantulan. Jurnal Kejuruteraan.

[19] Islam MR, Samsuzzaman M, Misran N, Beng GK, Islam MT (2020). A tri-band left-handed meta-atom enabled designed with high effective medium ratio for microwave based applications. Results Phys..

[20] Hussain N, Jeong M-J, Abbas A, Kim N (2020). Metasurface-based single-layer wideband circularly polarized MIMO antenna for 5G millimeter-wave systems. IEEE Access.

[21] Islam MS (2020). A gap coupled hexagonal split ring resonator based metamaterial for S-band and X-band microwave applications. IEEE Access.

[22] Moniruzzaman M (2020). Cross coupled interlinked split ring resonator based epsilon negative metamaterial with high effective medium ratio for multiband satellite and radar communications. Results Phys..

[23] Shah SMQA (2021). A multiband circular polarization selective metasurface for microwave applications. Sci. Rep..

[24] Almutairi AF (2019). A complementary split ring resonator based metamaterial with effective medium ratio for C-band microwave applications. Results Phys..

[25] Islam MS (2020). A mutual coupled concentric crossed-Line split ring resonator (CCSRR) based epsilon negative (ENG) metamaterial for Tri-band microwave applications. Results Phys..

[26] Abdulkarim Y (2020). Tunable left-hand characteristics in multi-nested square-split-ring enabled metamaterials. J. Cent. South Univ..

[27] Moniruzzaman M, Islam MT, Muhammad G, Singh MSJ, Samsuzzaman M (2020). Quad band metamaterial absorber based on asymmetric circular split ring resonator for multiband microwave applications. Results Phys..

[28] Hasan M-M (2022). Bilateral coupled epsilon negative metamaterial for dual band wireless communications. Comput. Mater. Continua.

[29] Srinivasan K, Ali NB, Trabelsi Y, Rajan MM, Kanzari M (2019). Design of a modified single-negative metamaterial structure for sensing application. Optik.

[30] Moniruzzaman M (2021). Inductively tuned modified split ring resonator based quad band epsilon negative (ENG) with near zero index (NZI) metamaterial for multiband antenna performance enhancement. Sci. Rep..

[31] Islam MR (2020). Square enclosed circle split ring resonator enabled epsilon negative (ENG) near zero index (NZI) metamaterial for gain enhancement of multiband satellite and radar antenna applications. Results Phys..

[32] Ramachandran T, Faruque MRI, Islam MT (2021). A dual-band polarization-independent left-handed symmetrical metamaterial for communication system application. J. Mater. Res..

[33] Al-badri KSL, Abdulkarim YI, Alkurt FÖ, Karaaslan M (2021). Simulated and experimental verification of the microwave dual-band metamaterial perfect absorber based on square patch with a 450 diagonal slot structure. J. Electromagn. Waves Appl..

[34] Hakim ML, Alam T, Almutairi AF, Mansor MF, Islam MT (2021). Polarization insensitivity characterization of dual-band perfect metamaterial absorber for K band sensing applications. Sci. Rep..

[35] Hannan S, Islam MT, Faruque MRI, Chowdhury ME, Musharavati F (2021). Angle-insensitive co-polarized metamaterial absorber based on equivalent circuit analysis for dual band WiFi applications. Sci. Rep..

[36] Bilal RMH (2021). Wideband microwave absorber comprising metallic split-ring resonators surrounded with E-shaped fractal metamaterial. IEEE Access.

[37] Abdulkarim YI (2021). A thermally stable and polarization insensitive square-shaped water metamaterial with ultra-broadband absorption. J. Mater. Res. Technol..

[38] Bait-Suwailam MM (2019). Electromagnetic Fields and Waves.

[39] Systemes, D. *CST STUDIO SUITE 2019*. https://www.3ds.com/products-services/simulia/products/cst-studio-suite/?utm_source=cst.com&utm_medium=301&utm_campaign=cst (2019).

[40] Chen X, Grzegorczyk TM, Wu B-I, Pacheco J, Kong JA (2004). Robust method to retrieve the constitutive effective parameters of metamaterials. Phys. Rev. E.

[41] Mahmoud AM, Engheta N (2014). Wave–matter interactions in epsilon-and-mu-near-zero structures. Nat. Commun..

[42] Wartak MS, Tsakmakidis KL, Hess O (2011). Introduction to metamaterials. Phys. Can..

[43] Hoque A (2018). A polarization independent quasi-TEM metamaterial absorber for X and Ku band sensing applications. Sensors.

[44] Zou H, Cheng Y (2019). Design of a six-band terahertz metamaterial absorber for temperature sensing application. Opt. Mater..

[45] Liu N, Giessen H (2010). Coupling effects in optical metamaterials. Angew. Chem. Int. Ed..

[46] Zhou H (2018). Multi-band sensing for dielectric property of chemicals using metamaterial integrated microfluidic sensor. Sci. Rep..

[47] Johnson MC, Brunton SL, Kundtz NB, Kutz JN (2015). Sidelobe canceling for reconfigurable holographic metamaterial antenna. IEEE Trans. Antennas Propag..

[48] Chuma EL, Iano Y, Fontgalland G, Roger LLB (2018). Microwave sensor for liquid dielectric characterization based on metamaterial complementary split ring resonator. IEEE Sens. J..

[49] Wu J (2018). Design and validation of liquid permittivity sensor based on RCRR microstrip metamaterial. Sens. Actuators A.

[50] Xiao Z-Y, Liu D-J, Ma X-L, Wang Z-H (2015). Multi-band transmissions of chiral metamaterials based on Fabry-Perot like resonators. Opt. Express.

[51] Bakır M, Karaaslan M, Unal E, Akgol O, Sabah C (2017). Microwave metamaterial absorber for sensing applications. Opto-Electron. Rev..

[52] Liu S-H, Guo L-X, Li J-C (2016). Left-handed metamaterials based on only modified circular electric resonators. J. Mod. Opt..

[53] Idrus IN (2021). An octagonal split ring resonator-based double negative metamaterial for S-, X-and Ku-band applications. Proc. Inst. Mech. Eng. Part L J. Mater. Des. Appl..

